# An Atlas of the Thioredoxin Fold Class Reveals the Complexity of Function-Enabling Adaptations

**DOI:** 10.1371/journal.pcbi.1000541

**Published:** 2009-10-23

**Authors:** Holly J. Atkinson, Patricia C. Babbitt

**Affiliations:** 1 Graduate Program in Biological and Medical Informatics, University of California, San Francisco, California, United States of America; 2 Institute for Quantitative Biosciences, University of California, San Francisco, California, United States of America; 3 Department of Bioengineering and Therapeutic Sciences, University of California, San Francisco, California, United States of America; 4 Department of Pharmaceutical Chemistry, University of California, San Francisco, California, United States of America; Fox Chase Cancer Center, United States of America

## Abstract

The group of proteins that contain a thioredoxin (Trx) fold is huge and diverse. Assessment of the variation in catalytic machinery of Trx fold proteins is essential in providing a foundation for understanding their functional diversity and predicting the function of the many uncharacterized members of the class. The proteins of the Trx fold class retain common features—including variations on a dithiol CxxC active site motif—that lead to delivery of function. We use protein similarity networks to guide an analysis of how structural and sequence motifs track with catalytic function and taxonomic categories for 4,082 representative sequences spanning the known superfamilies of the Trx fold. Domain structure in the fold class is varied and modular, with 2.8% of sequences containing more than one Trx fold domain. Most member proteins are bacterial. The fold class exhibits many modifications to the CxxC active site motif—only 56.8% of proteins have both cysteines, and no functional groupings have absolute conservation of the expected catalytic motif. Only a small fraction of Trx fold sequences have been functionally characterized. This work provides a global view of the complex distribution of domains and catalytic machinery throughout the fold class, showing that each superfamily contains remnants of the CxxC active site. The unifying context provided by this work can guide the comparison of members of different Trx fold superfamilies to gain insight about their structure-function relationships, illustrated here with the thioredoxins and peroxiredoxins.

## Author Summary

For any large class of proteins, far more protein sequences are known than can be examined experimentally. This is the case with the thioredoxin fold class, a large and diverse collection of proteins, some of which are known to catalyze important steps in metabolism. Some others participate in key processes like protein folding and detoxification of foreign compounds. Many of the unstudied proteins likely participate in other important biological processes and have useful applications in medicine and industry. We used a new network-based computational approach to create similarity-based maps of the thioredoxin fold class. These maps juxtapose unstudied proteins with similar well-characterized proteins, helping to show where existing knowledge can help predict properties of uncharacterized sequences. This information can be used to identify which of these sequences are interesting and deserve experimental characterization. We also used the maps to gain insight about how shared structural features are used and modified to affect catalysis in the different subclasses, leading to a better understanding of the interplay between structure and function in the thioredoxin fold class.

## Introduction

It has been established that protein structures incorporate new variations on an ancestral fold in evolving diverse functions [1]. Domains recombine in modular units, are decorated with insertions and extensions of loops and secondary structure elements [2], and sometimes they drift [3]. However, *how* these large revisions to a fold can extend and transform the catalytic capabilities of a protein is less understood for a number of reasons, namely that the catalytic changes are system-specific and that trends can often only be detected through observing the full landscape of variations of the fold. As more new proteins are discovered that are united principally by distant similarities in fold and active site machinery, it becomes more important to leverage knowledge of their structure-function relationships in order to ask targeted questions about their potential functions. Knowledge of the interplay between fold variation and function can suggest assays for *in vitro* and *in vivo* molecular function and biological roles.

The thioredoxin fold class is a prime example of why such a clarification is desirable; members evince extreme levels of structural and functional variation when compared with the canonical thioredoxin enzyme. The class (or group, as distinct from the term ‘class’ as it is used in structural biology, which refers to secondary structure composition) comprises a broad collection of protein superfamilies that are unified by their shared use of the small thioredoxin (Trx) domain—consisting of a four-stranded beta sheet sandwiched by three alpha helices—and diversified by the many molecular functions catalyzed by members of the fold class (see Table 1 and reviews referenced therein; described in [4],[5]). Trx fold proteins are found in every organism, playing critical roles in defense from oxidative stress [6], protein folding [7], and enzymatic detoxification of xenobiotics [8], but only 5.6% of Trx fold proteins have been manually associated with a functional annotation of any type. (5.6% of Trx fold proteins analyzed in this work are annotated in the hand-curated SwissProt database; the remainder is found in the TrEMBL database [9].) Through decades of extensive experimentation with a subset of Trx fold proteins, it is known that many of these enzymes are medically important. For example, defects in some of these proteins are implicated in human disease, including cancer and Alzheimer's Disease (e.g., [10],[11]), and other Trx fold proteins in infectious organisms are targeted in drug development efforts (e.g., [12]). However, as shown in this work, it is clear that the well-studied proteins are only a small sampling of the structural and functional diversity present in the larger Trx fold class.

**Table 1:** Typical molecular functions of major Trx fold superfamilies.

Thioredoxin (Trx)	Reduction of disulfide bonds in proteins [6]
Glutathione peroxidases (GSHPx)	Reduction of hydroperoxides [71]
Peroxiredoxins (AhpC-TSA, Redoxin, Prx)	Reduction of hydroperoxides [60],[72]
Sco (SCO1-SenC)	Copper ion binding; thiol-disulfide oxidoreductase activity [31]
Dsb (DSBA)	Formation of disulfide bonds in proteins [7]
ArsC	Reduction of arsenate [73]
Glutaredoxin (Grx)	Reduction of disulfide bonds in proteins; deglutathionylation of proteins [14],[70]
Glutathione transferase (GST, GST_N)	Addition of glutathione to small molecules; reduction of hydroperoxides [8]

**Figure 1:**
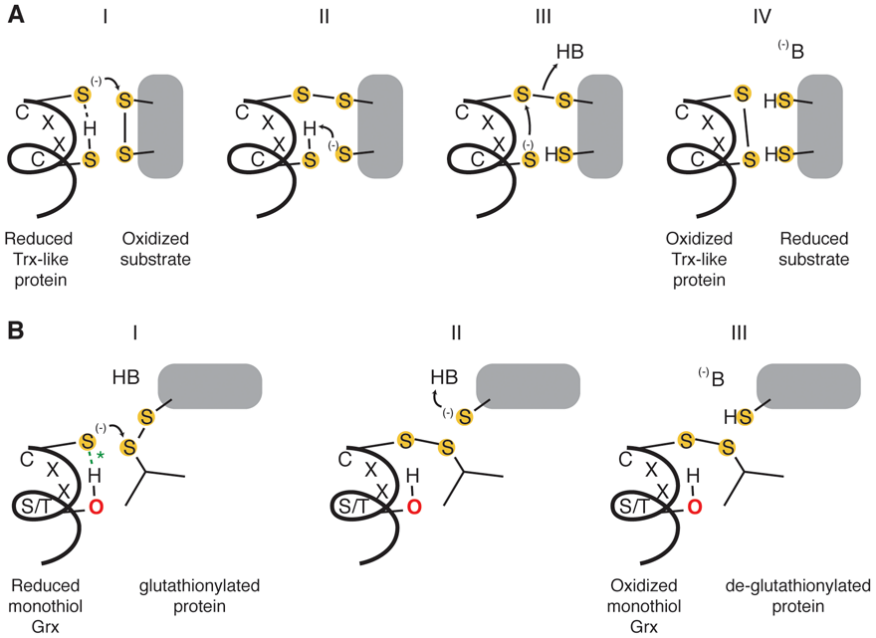
Dithiol and monothiol Trx fold reactions. **A** The archetypal thioredoxin reaction, entailing the reduction of a disulfide bond by a thioredoxin-like protein equipped with a dithiol CxxC active site. **B** The reduction of a mixed disulfide bond between glutathione and a protein by a monothiol glutaredoxin (Grx). In step I, the interaction between the hydroxyl hydrogen of a serine or threonine (green *) is suggested by conserved sequence motifs. Key: B denotes a general base. (Adapted from [70].).

**Figure 2:**
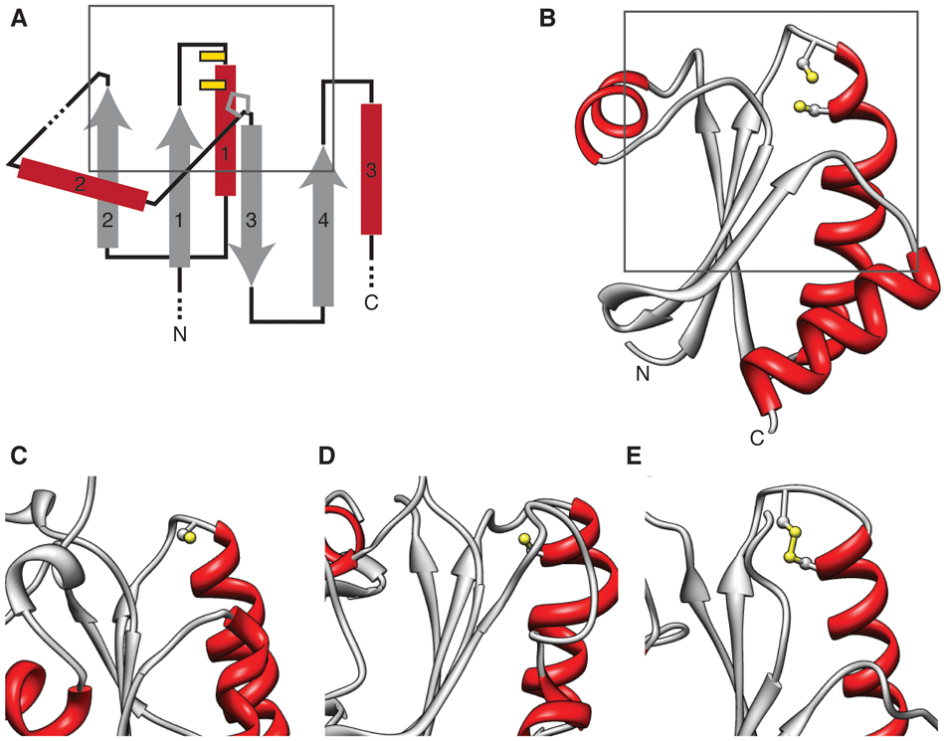
Most Trx fold active sites involve catalytic cysteines. **A** A topological diagram of the Trx fold, showing the four-stranded mixed beta sheet sandwiched by three alpha helices. The archetypal CxxC active site cysteines from thioredoxin are represented by yellow bars near the N-terminus of the first alpha helix. Also shown are common locations for insertions and extensions relative to the Trx fold (dashes), and the position of a *cis*-proline that is frequently found at the N-terminus of the third beta strand. A grey box denotes the region of the fold shown in C–E. Active site types are abbreviated using a motif like “CxxC”, where a ‘C’ indicates presence of a cysteine, and ‘c’ indicates the presence of some residue other than cysteine. “CxxxC” means the active site cysteines are separated by three amino acids. **B** The classic CxxC active site, illustrated by human Trx 2 (PDB:1UVZ); Cys 31 and Cys 34 are shown. A grey box denotes the corresponding region of the fold shown in C–E. **C** The Cxxc active site, where the second cysteine has been mutated to another residue, illustrated by *E. coli* ArsC (PDB:1I9D); Cys 12 is shown (active site: CxxS). **D** The cxxC active site, in which the N-terminal Trx Cys has been lost, illustrated by human peroxiredoxin 5 (PDB:1OC3); Cys 47 is shown (active site: TxxC). **E** The CxxxC active site, in which the N-terminal Cys has been shifted further into the loop between the first beta strand and alpha helix, illustrated by *S. cerevisiae* SCO1 (PDB:2B7J); a disulfide bond between Cys 148 and Cys 152 is shown.

Beyond the basic commonality of the Trx domain, class members are linked by a distribution of remnants of the canonical active site and catalytic mechanism. The archetypal catalytic mechanism in the Trx fold class involves the reduction of a disulfide bond in a protein substrate using a dithiol CxxC active site [6] (Fig. 1A). This motif is very common in the Trx fold class, but is by no means ubiquitous. At a basic level, variations on the canonical CxxC motif can be reduced to four categories based on the number and positioning of cysteine residues known to be involved in the catalytic mechanism (Fig. 2). The first cysteine of the canonical CxxC motif of thioredoxin provides a nucleophilic thiolate positioned at the N-terminus of an alpha helix. In the canonical thioredoxin reaction, a disulfide bond is reduced in a substrate protein, and the necessary nucleophilic thiolate is partly stabilized by proton sharing between the N- and C-terminal cysteine thiols [13]. However, only a single cysteine is implicated in the reactions of certain Trx fold superfamilies (e.g. [14]), and some members of the fold class have retained none of the archetypal pair of cysteines. Some of these cysteine-less proteins are catalytic (e.g., [8]), and some are not (e.g., [15]). For the former, this begs the question of how the Trx fold itself facilitates oxidoreductase and other reactions in the absence of the standard catalytic equipment.

As more diverse members of the Trx fold class have come to light, a number of analyses have revealed important trends that characterize the class. Fomenko and Gladyshev analyzed CxxC motif variations in different types of Trx fold proteins, linking different CxxC-derived motifs to homologous and non-homologous proteins with oxidoreductase function to estimate the occurrence frequency of each motif in four model organisms [16],[17]. Qi and Grishin provided a comprehensive accounting of the types of structurally characterized proteins containing the thioredoxin fold [4]. Kortemme and Creighton assessed the contribution of the local secondary structural environment to the stabilization of the nucleophilic thiolate in the CxxC motif using model peptides [18]. However, there have not been any systematic analyses of the representation of Trx fold proteins in different phylogenetic categories outside of a few kingdom-specific analyses for individual superfamilies or families (e.g., plant GSTs [19] and parasite peroxiredoxins [20]). While these analyses of specific types of Trx fold proteins are useful, they do not establish a global picture of variation across the entire fold class. Also missing in the available large-scale analyses is a discussion of the molecular functions enabled by variations of the Trx fold and how similar one version of the Trx fold is to another. In the Trx fold class as well as other enzyme super- and suprafamilies [21], ultimately, we lack a fundamental theory of how intrinsic structural elements of a given fold enable function. The development of such a theory could provide a roadmap for efforts in enzyme annotation, engineering and drug targeting [22].

In this work, we attempt to address these questions by identifying some of the underlying themes in how the thioredoxin fold scaffold has been modified through evolution to enable a wide variety of functions, assisted by our use of a new network-based approach for analyzing large collections of proteins. Realizing that diverse members of the Trx fold class retain common features, both fold dependent and sequence dependent, that lead to delivery of function [23],[24], here the class has been treated as a single functionally distinct suprafamily as defined by Gerlt and Babbitt [21], i.e., a set of divergently related enzymes whose members catalyze different overall reactions that do not share a common mechanistic strategy. This work uses protein similarity networks [25], in which proteins are represented as nodes in a network connected by similarity information drawn from pairwise structural or sequence comparisons. The resulting networks are used to directly visualize information about function, sequence motifs, and species taxonomy for 159 structures and 4,082 sequences spanning the full Trx fold class. Although we use representative sequences and structures, this atlas comprises the largest set of Trx fold proteins that has been considered to date, and it incorporates data from recent genome and structural genomics initiatives which are often overlooked in investigations of more familiar proteins [26]. We have attempted to clarify the relative similarity between the major classes of Trx fold proteins by using protein similarity networks to show how the different superfamilies of the fold class are related by structure and sequence. We also present a map of the prevalence of Trx fold superfamilies across kingdoms of life and the distribution of different catalytic motifs throughout the Trx fold. The resulting landscape, combining structural similarity with clues for inferring molecular function, provides a framework for comparing members of different superfamilies, a key task for querying their structure-function relationships and enabling functional annotation for the unknown proteins on the fringes of the thioredoxin fold class.

## Results/Discussion

In the following sections, we describe how we use information first from structures and second from sequences spanning the entire Trx fold class to observe structural and functional relationships between member superfamilies, as well as to understand how their different functions are accomplished using varied and modular domain structures. The third section canvasses the populations of each superfamily to demonstrate which oxidoreductase strategies are used by different organisms in the Tree of Life. The fourth section reveals the diversity in implementations of some of the most fundamental aspects of catalysis for each type of thioredoxin fold domain, while the last section uses the full thioredoxin fold context to present a new view of the relationship between the classical thioredoxins and the peroxiredoxins. A figure summarizing the results is provided in the second section.

A note on nomenclature: We attempt to follow the suprafamily-superfamily-subgroup-family hierarchy outlined in Gerlt and Babbitt, 2001 [21], using the phrase “group” or “class” when the granularity of functional annotation is unclear. We frequently refer to groups of protein termed as families by PFAM [27], which generally correspond to our definition of *superfamily*, as well as the PFAM Thioredoxin-like Clan [28], which is equivalent to our definition of the thioredoxin *suprafamily*. A *superfamily* is a group of homologous enzymes that catalyze either (a) the same chemical reaction with differing substrate specificities or (b) different overall reactions that conserve a subset of active site residues that perform the same mechanistic roles. A *suprafamily* is a group of homologous enzymes that catalyze different overall reactions but whose reactions do not share common mechanistic attributes. Although active site residues may be conserved, these perform different functions in the members of the superfamily. As members of the thioredoxin fold class are thought to be evolutionarily related, the fold class is also a suprafamily. A *subgroup* is a classification that falls between family (in which all members catalyze the same reaction in the same way) and superfamily; this is typically based on sequence-based clustering. This work does not describe functional annotations for groups of proteins more specific than the subgroup level: as a broad overview of the thioredoxin fold, without additional experiments, we cannot label all sequences with specificity annotations, or sometimes even reaction class, because too little is known about the *in vitro* or *in vivo* function of large expanses of the fold class. Following historical convention, the thioredoxin superfamily and thioredoxin fold class/suprafamily are named for the thioredoxin protein.

### Structures of the thioredoxin fold class show how the constituent superfamilies are related by structural similarity

**Figure 3:**
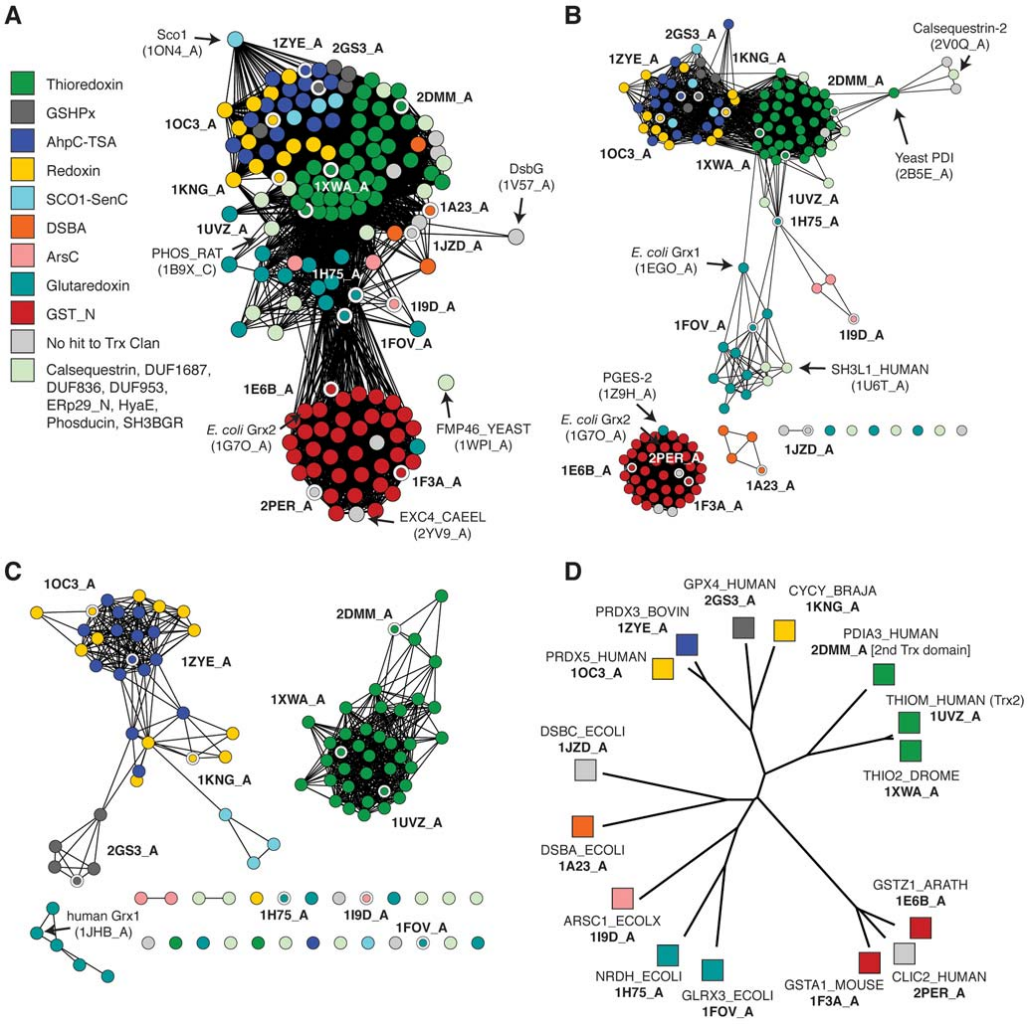
A structure-based similarity network describes a map of the Trx fold class. A Structure similarity network, containing 159 structures that are a maximum of 60% identical (by sequence) that span the Trx fold class. Similarity is defined by FAST scores better than a score of 4.5; edges at this threshold represent alignments with a median of 2.75 Å RMSD across 72 aligned positions, while the rest of the edges represent better alignments. As given in the key, each node is colored by a PFAM Thioredoxin-like Clan family if the chain sequence is a member. (Non-members are colored grey and labeled “No hit to Trx Clan.) These classes are discussed briefly in Table 1. Nodes with thick white borders and bold labels denote chains present in the hierarchical clustering tree in D. Labels like “1ON4_A” denote PDB ID 1ON4, chain A. Some additional proteins that may be of interest are labeled with plain face text and labels. B Structure similarity network containing the same structures as in A, shown at the more stringent threshold of 7.5. Edges at this threshold correspond to alignments with a median of 2.45 Å RMSD across 89 aligned positions. Nodes are colored as in A. C Structure similarity network containing the 105 structures from the large connected cluster in B, displayed at a FAST score cutoff of 12.0; edges at this threshold represent alignments with a median of 2.21 Å RMSD across 102 aligned positions. Nodes are colored as in A. D Complete linkage hierarchical clustering tree based on pairwise FAST scores for 15 representative structures singled out in the networks in A–C, with PDB IDs in bold, and associated SwissProt sequence IDs in plain text. Note: this is a static figure generated from interactive protein similarity networks that can be downloaded and viewed from http://babbittlab.compbio.ucsf.edu/resources/TrxFold/.

Global trends in structural similarity between different variants of the thioredoxin fold can be visualized using a similarity network, in which nodes represent chains from experimentally determined structures, and edges connecting nodes represent 3D similarity relationships better than a threshold. The lengths of edges in the network are strongly correlated with similarity between the pairs of proteins: in general, the shorter the edge connecting two proteins, the more similar the pair of proteins [25] (see Fig. 3). Different degrees of sequence similarity can be emphasized by varying the threshold score, for example in Fig. 3A, distant relationships are included, emphasizing superfamily-level groupings, while in Fig. 3C, the threshold is more stringent and only the most similar protein structures are connected. Disconnected proteins and clusters might be related by detectable sequence similarity at levels below the selected threshold score. These disconnected proteins typically appear in rows at the bottom of a similarity-network-based figure, and their location relative to other groups is arbitrary.

As might be expected, some of the large-scale trends in structural similarity are paired with similarity in catalytic function. By viewing the structural similarity relationships at more stringent thresholds, finer distinctions emerge, showing that peroxiredoxins (Redoxin, AhpC) and glutathione peroxidases (GSHPx) are more similar to one another than to thioredoxins (Fig. 3B–C). One of the most common modifications to the Trx fold is an insertion of secondary structure elements between the second beta strand and the second alpha helix (Fig. 2A). In this case, all peroxiredoxins, and glutathione peroxidases have an alpha helix-beta strand insertion at that position [5]. This additional structural similarity between peroxiredoxins and GSHPxs is important—despite being considered different superfamilies, both groups solely catalyze reductions of hydroperoxides, although GSHPxs are known to be far more efficient, particularly GSHPxs with selenocysteine active sites [29]. Likewise, although they catalyze different reactions than those of the peroxiredoxins and GSHPxs, the cytochrome maturation proteins (CMP; see 1KNG in Fig. 3) also have this structurally similar helix strand insertion, much like its heretofore-undescribed appearance in the Sco1-like proteins. CMPs (variously known as CcmG, DsbE, cycY, ResA, and others) are associated with the reduction of apocytochrome C in bacteria [30], while human Sco1 is known to function further down the electron transfer chain in the maturation of cytochrome C oxidase [31]. Two other superfamilies of enzymes with insertions in the same region of the Trx fold are the DsbA-like enzymes and ArsC. The insertions in both of these groups are quite different (with respect to the CMP insertion, as well as to one another) and large, consisting of four to five alpha helices replacing the second helix of the Trx fold. The DsbA and ArsC insertions are also oriented differently with respect to the Trx fold. Based on the census provided in this work, it appears that this is the only region of the Trx fold that can easily tolerate an insertion. The only other major structural modification to the Trx fold is the presence of additional domains before and after the complete Trx fold.

The network topology also demonstrates that glutaredoxins (Grx) are not a cohesive superfamily, an idea that is supported by many reports from the literature. First, the structure-based network shows that Grxs are quite structurally diverse. This may be a consequence of a deficiency in sampling of their structures; as a group they are only loosely connected, indicating fewer similarity relationships better than the thresholds in Fig. 3. In particular, *E. coli* Grx 2 (1G7O) is structurally most similar to the glutathione transferases (GST), as reported earlier [32]. In fact, it is a distant GST superfamily member, exhibiting faint but identifiable sequence similarity across the length of the complete GST domain despite its classic dithiol Grx CPYC active site motif and glutaredoxin activity (see Fig. 3A,B). Indeed, the definition of a glutaredoxin is somewhat pliable; classically, glutaredoxins are proteins that reduce disulfide bonds and are recycled via glutathione disulfide and glutathione reductase [33]. Yet a number of apparent glutaredoxins have been shown to behave like thioredoxins, serving as substrates for thioredoxin reductase [34]–[40]. (The proteins in these examples are typically annotated as glutaredoxins on the basis of having a CPYC motif or being a better match to the PFAM Glutaredoxin family model than the Thioredoxin model.) Consider also the omega class GSTs that demonstrate glutaredoxin activity *in vitro* [41],[42], and the GST superfamily member *E. coli* yfcG, which has low activity on model GST substrates but efficiently catalyzes a model glutaredoxin reaction [43]—the term glutaredoxin may in fact be an umbrella term for a number of enzyme superfamilies demonstrating a common *in vitro* catalytic capability, yet that are no more related than any other pair of superfamilies in the Trx fold with respect to their structural similarity and roles in metabolism. Glutaredoxins share additional unusual qualities; as a class, they exhibit an enhanced level of domain modularity and flexibility in their active site motif relative to other thioredoxin-like superfamilies, as will be discussed further in the following sections.

When studied individually, many new and distant Trx fold class members have been discussed as outliers relative to the nearest superfamily. Some of these minority enzyme superfamilies and families can be placed into the broader context of the suprafamily using the structural network (Fig. 3). When viewed from the context of the global Trx structural landscape, it becomes clear that there are different degrees of structural outlier status within the fold class. For example, the human and *C. elegans* chloride intracellular channel (CLIC) proteins (2PER and 2YV9) are tightly grouped with the GSTs, and calsequestrin is most similar to the classic thioredoxin superfamily, as are the ER-localized proteins rat ERP29 and *D. melanogaster* windbeutel. The Trx domain in rat phosducin (1B9X_C) can only be related to the rest of the Trx Clan structures at relatively low levels of similarity (Fig. S1; Fig. S1 shows nodes colored by the minority families that are not distinguished in Fig. 3). See Table S1 for an accounting of the number of unique structures in each thioredoxin fold member superfamily. The trends evident from the structural network topology are mirrored in a tree demonstrating a hierarchical clustering of fifteen representative structures from the similarity network (Fig. 3D).

### A sequence similarity map of the thioredoxin fold class illustrates diversity in function and in domain structure

**Figure 4:**
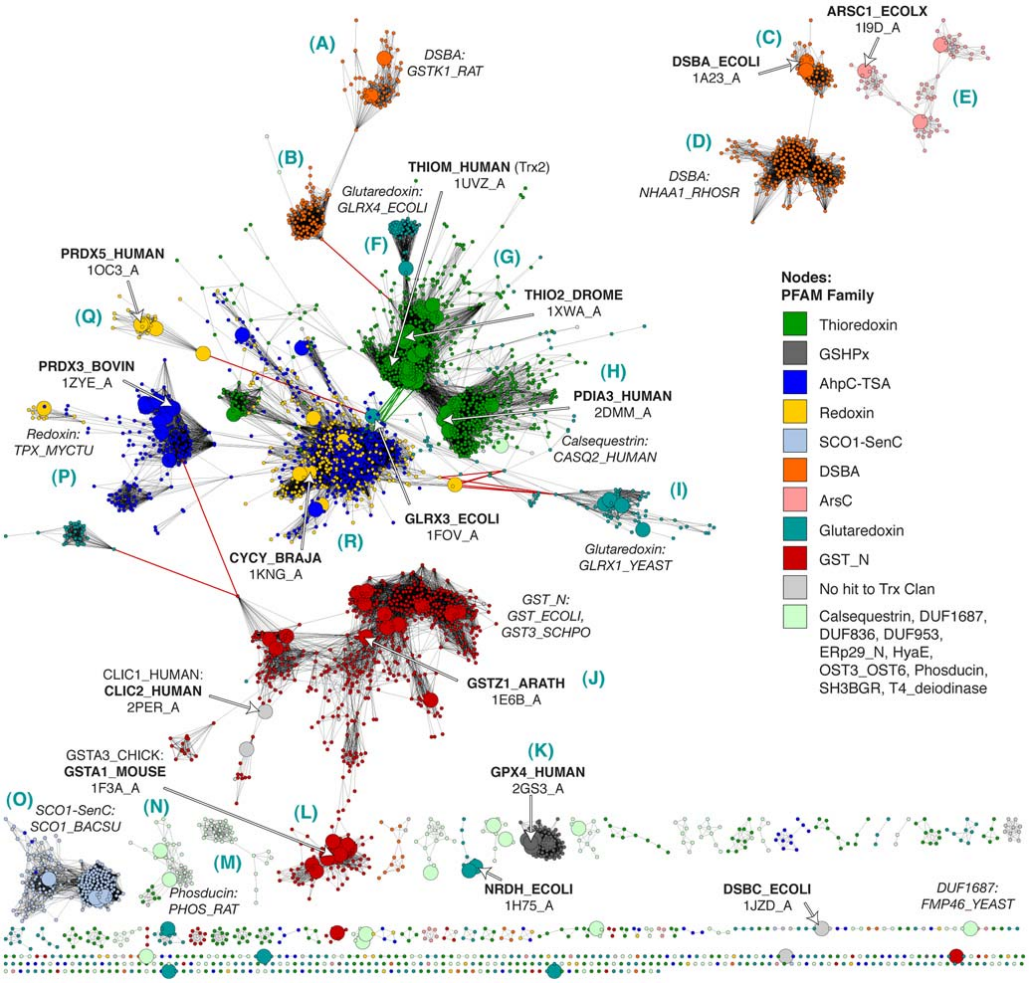
A sequence similarity network shows how each Trx fold superfamily is distributed. Sequence similarity network, containing 4,082 representative sequences that are a maximum of 40% identical and span the Trx fold class. Similarity is defined by pairwise BLAST alignments better than an E-value of 1×10^−12^; edges at this threshold represent alignments with a median 30% identity over 120 residues, while the rest of the edges represent better alignments. Each node is colored by a PFAM Thioredoxin-like Clan family if the sequence is a member. (Non-members are colored grey and labeled “No hit to Trx Clan.) These classes are discussed briefly in Table 1. Large nodes represent sequences that are associated with the 159 structures in Fig. 3. The sequences associated with the 15 representative structures in Fig. 3C are labeled using bold text and white arrows. The general locations of other sequences representing different superfamilies are noted using italicized text. Some edges representing similarity relationships from outside of the domain of interest are colored red, and are discussed in the text. Blue letters in parentheses correspond to the labels defining each group in Figures 5–7.

The distant similarity relationships between and within Trx fold superfamilies are best shown using structural similarity. However, finer relationships that enhance the observation of the interplay between primary structure and function can be discerned by viewing many sequences representing the full breadth of the Trx fold class as a larger, more detail-rich sequence similarity network. In contrast to the networks in Fig. 3, which incorporate extremely distant structure-based relationships to accentuate similarities between variations of the Trx fold, the sequence similarity network in Fig. 4 shows 4,082 representative sequences from the Trx fold class that are clustered on the basis of pairwise sequence alignments. The most distant of these alignments are roughly significant enough to highlight superfamily-level groupings and major classes within superfamilies. The greater sequence coverage and finer distinctions between groups that are revealed by the network topology yield a unique, “30,000-foot-view” of class biases at play within the thioredoxin fold suprafamily.

**Figure 5:**
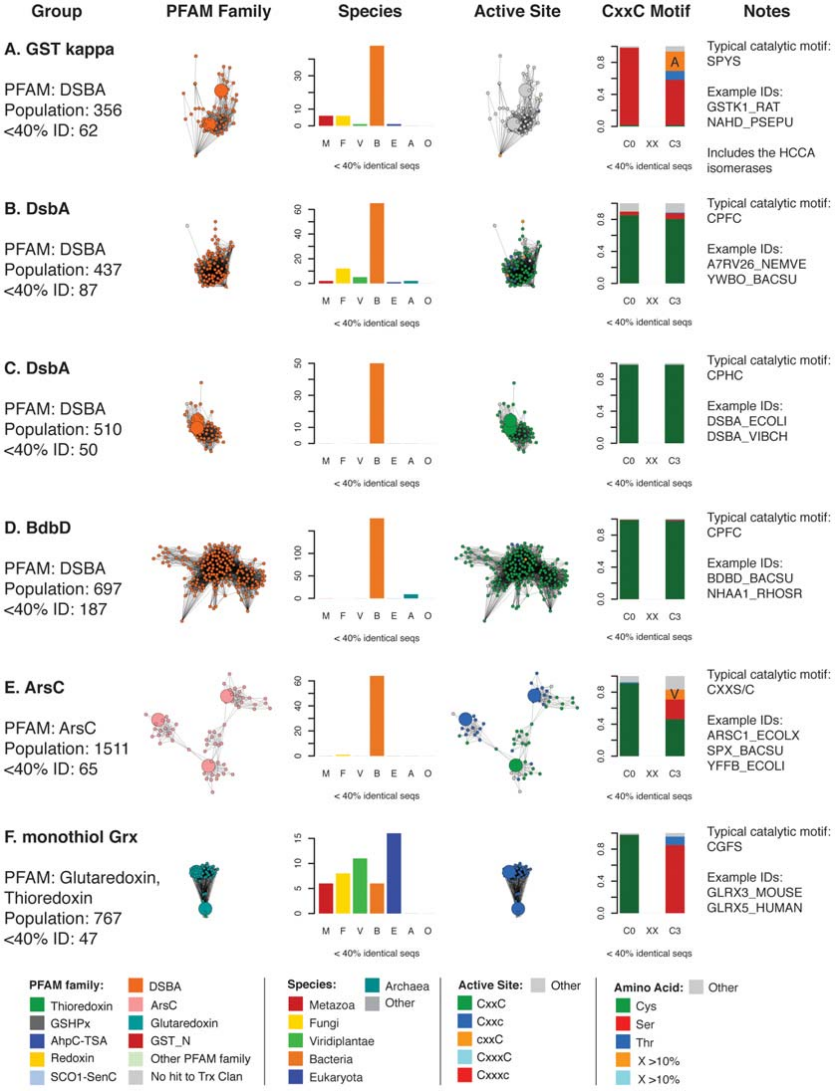
Summary of taxonomic and active site motif properties for Trx fold sequence groups (A–F). Selected sequence classes marked with blue letters in Fig. 4 are summarized here. Coloring varies in the four columns of networks and bar charts—each is colored differently according to the legend at the bottom of each figure. Listed are: **Group:** the most prevalent PFAM family classification[s], the population without sequence filtering (“Population”) and the population after filtering to a maximum of 40% identity as shown in the adjacent network excerpt (“<40% ID”). See Table S4 for the mapping between these groups and the databases PFAM [27], SCOP [68], and CATH [69]. PFAM **Family:** the network cluster excerpted from Fig. 4. **Species:** a bar chart showing the distribution of species categories among sequences from the network; note that “Eukaryota” includes all eukyaryotic species without a more specific kingdom, and is primarily associated with protozoan parasites. **Active Site:** the network cluster colored by predicted active site architecture; these clusters are excerpted from Fig. 8. CxxC means both active site cysteines are present, Cxxc means only the N-terminal cysteine is present, cxxC implies the presence of the C-terminal cysteine, CxxxC indicates that there are three positions between the two cysteines, and “Other” means that neither cysteine is present in the expected position. **CxxC Motif:** a bar chart indicating the type of residue substitutions at the two key positions of the CxxC motif for that group. The stacked bars include the fraction of active sites incorporating a Cys, Thr, or Ser, as well as any other amino acid occurring more than 10% of the time (orange and light blue in key). Otherwise, residues other than cysteine, threonine, or serine are included in the grey “Other” category. **Notes:** column lists an example high-frequency CxxC motif and example UniProt IDs for sequences in the group.

**Figure 6 pcbi-1000541-g006:**
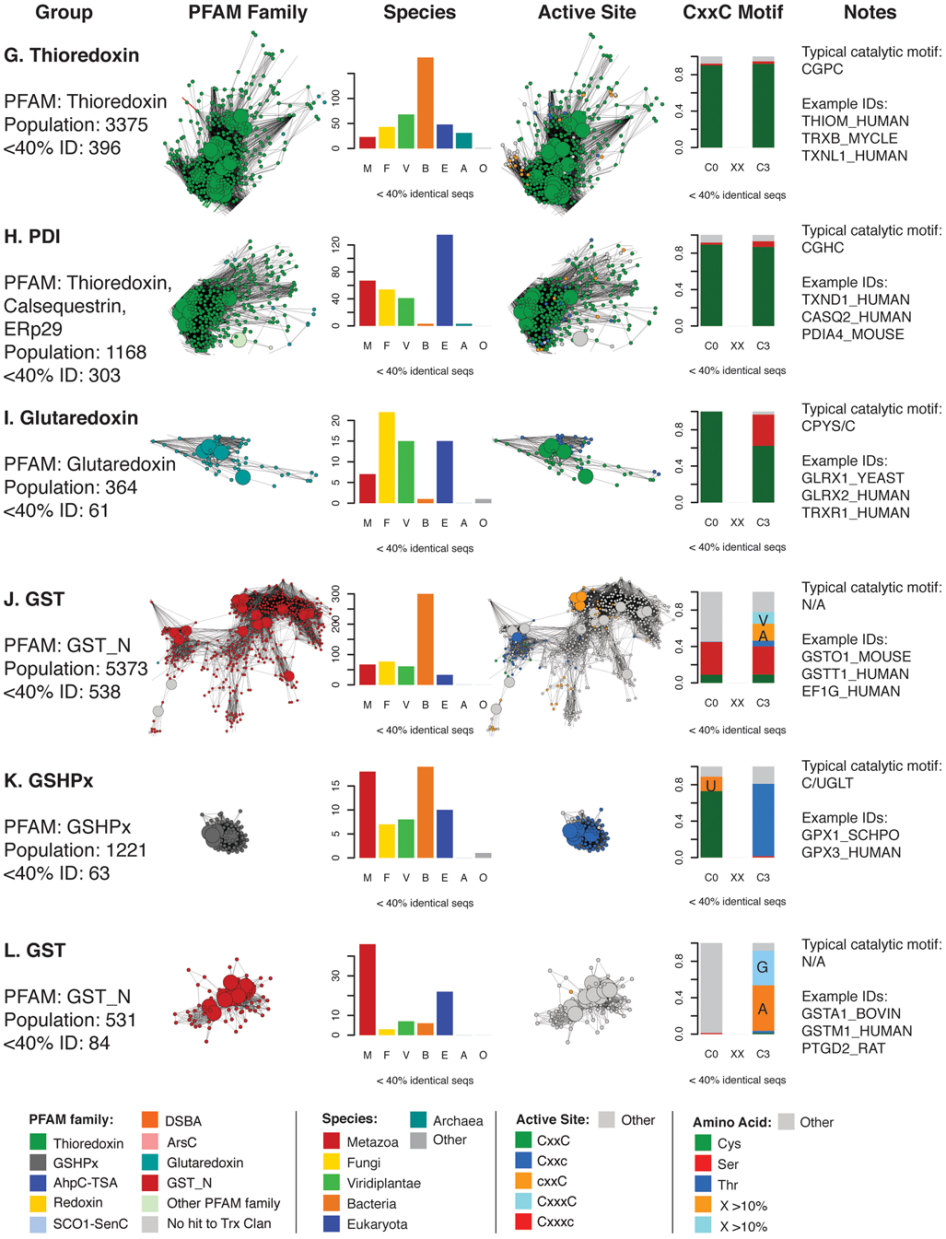
Summary of taxonomic and active site motif properties for Trx fold sequence groups (G–L). See Figure 5 legend.

Rather than separating into two major classes of GST-like and Trx-like as in Fig. 3A, the sequence similarity network in Fig. 4 reveals a large number of clusters, most of which correspond to known functional classes (compare Fig. 4, with nodes annotated by PFAM family membership, to Fig. S2, with nodes annotated by SwissProt family classifications). Information about these clusters of proteins is summarized in Figures 5–7. As the equivalently colored proteins in the structure networks in Fig. 3 show, when much more distant levels of similarity are included, like colors (superfamilies) will be grouped together in the network. (The exceptions are the Redoxin and AhpC PFAM families, as the models describing these families overlap, and the Glutaredoxin family, which is genuinely heterogeneous.) While the individual thioredoxin-like domains in the classic thioredoxin and protein disulfide isomerases (PDI) are structurally very similar (Fig. 3), they form two distinct groups at the level of sequence similarity (Fig. 6G,H). This co-occurs with a functional expansion from reduction of disulfide bonds (thioredoxin) to oxidation and isomerization of disulfide bonds (PDI). Echoing the patterns in the structural network, the glutaredoxins form many discrete clusters that are disconnected at this similarity cutoff of E = 10^−12^ (thirty percent sequence identity over alignments of 120 residues). The monothiol glutaredoxins (Fig. 5F) are generally distant from other classes of glutaredoxins, and the *E. coli* Grx4/human Grx5 monothiol glutaredoxins are joined with the thioredoxin group via an N-terminal thioredoxin domain embedded in each sequence. These proteins have been recently associated with a number of diverse and specific biological functions, including iron-sulfur cluster biogenesis and regulation of cardiac function [14], which are quite distinct from the classic glutaredoxin role as a general disulfide reductase. Many of the clusters of sequences in Fig. 4 are associated with a shift to a new phylogenetic profile within a superfamily, such as the two groups of GSTs (Fig. 6J,L), and the DsbA-like proteins containing GST kappa (Fig. 5A), and will be discussed further in the following section.

**Figure 7 pcbi-1000541-g007:**
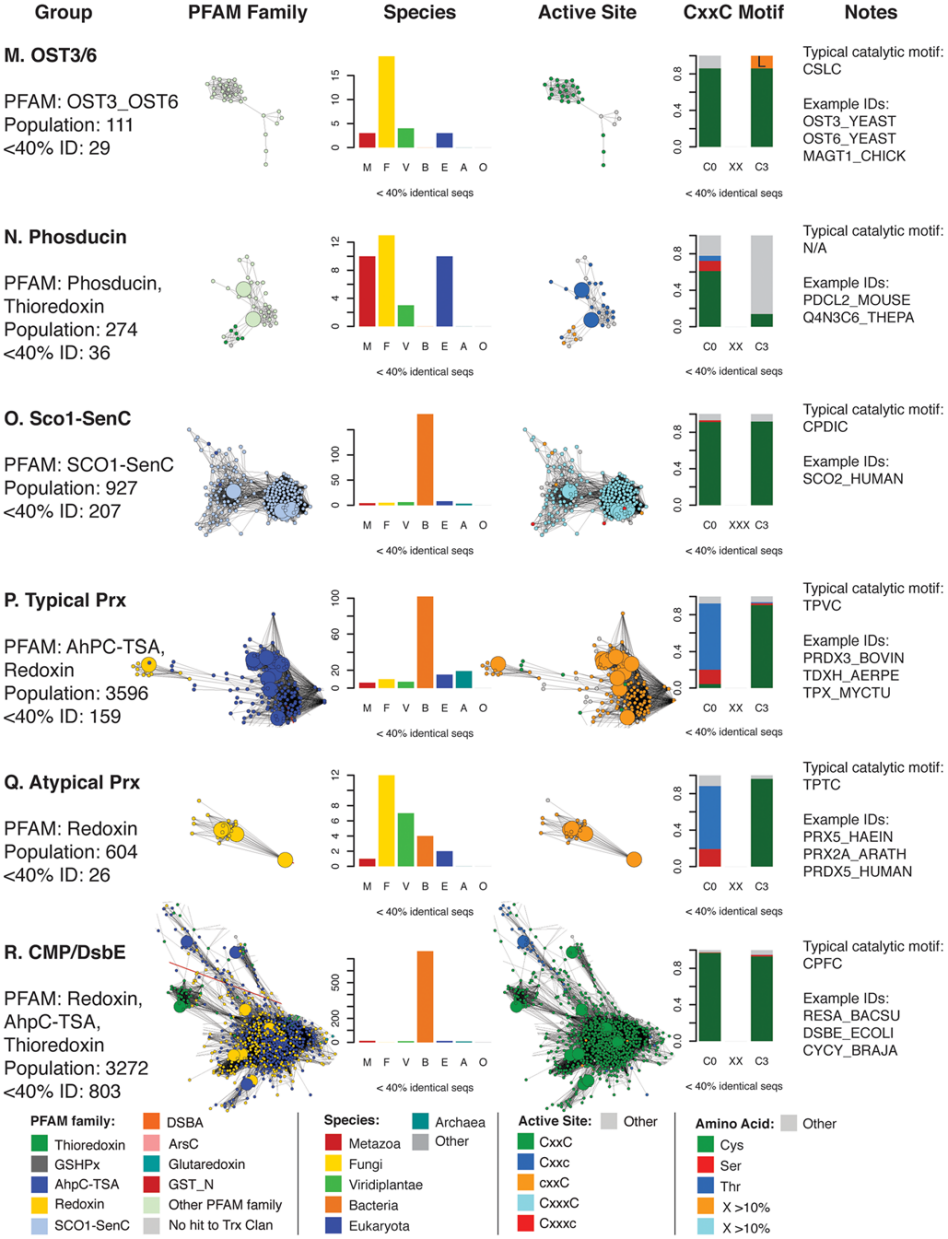
Summary of taxonomic and active site motif properties for Trx fold sequence groups (M–R). See Figure 5 legend.

The protein domain structure within the Trx fold class is varied and modular. Analysis of these sequences indicates that while most members contain just one copy of a certain thioredoxin fold domain embedded in the protein-coding sequence, some classes typically contain multiple copies (see Fig. S3); 2.8% of the 4,082 sequences depicted in Fig. 4 contain two to four domains from the Trx fold class. A number of bacterial DsbA-like sequences contain two or three PFAM DSBA domains (in Fig. 5D), and certain monothiol glutaredoxins pair a thioredoxin domain with one, two, or three glutaredoxin domains (in Fig. 5F). Protein disulfide isomerases are known to contain multiple thioredoxin domains; in this analysis, PDI-like proteins are seen to contain anywhere from one to four thioredoxin domains in sequence. Some of the variation in PDI proteins is thought to be necessary for enabling different substrate specificities [44]. Interestingly, only the glutaredoxin domain was found in combination with any other Trx fold domain, as in the example of the fused Prx 5/glutaredoxin in *H. influenzae* (in Fig. 7Q). The crystal structure of *H. influenzae* Prx 5 shows how these two domains may interact in other organisms in which the domains are not fused [45]. Another aspect of domain modularity in the Trx fold class is the presence of additional domains in the protein-coding sequence, such as a kinase domain, from outside of the Trx fold. The quiescin-sulfydryl oxidases (QSOX), which cluster with the PDI-like proteins and are thought to participate in oxidative protein folding, pair two Trx domains with a non-Trx flavin-binding domain that provides an intermediate electron acceptor [46]. Variants of oxidoreductase activity are important in metabolism, especially respiration, and these domain combinations can provide clues to where Trx fold proteins are involved in sequences of metabolic events. A small set of edges displayed in the network in Fig. 4 are due to similarity between non-Trx domains and are colored red (detailed in Table S3).

Notably, outside of proteins consisting of a single domain, the majority of any superfamily or large sequence similarity group shares no specific multidomain structure. The sequence of a single domain protein embedded in the network among other PDI-like proteins can be quite similar to an individual domain within a 2-, 3-, or 4-domain PDI-like protein. As all four Trx-like domains present in yeast PDI are necessary for its role as a foldase [47], the existence of many similar domains in isolation hints toward many undiscovered complexes and physiological roles for proteins in this class.

Finally, this analysis has revealed some general features of the Trx fold class. The different member superfamilies have vastly different populations and represent different levels of sequence diversity. The classic thioredoxin superfamily (as distinguished from the thioredoxin fold class) represents the largest contribution to the sequence diversity of the whole fold class (Fig. S4A), whereas the GST-like enzymes are populated by more known sequences than any other superfamily in the fold class (Fig. S4B, Table S2). Additionally, by viewing the sequences associated with structures from Fig. 3 mapped to the sequence network in Fig. 4, it is clear that the Trx fold class has good structural coverage, despite the high ratio of sequences to available structures (see also Table S1). There is also good correspondence between the information in the structure-based network and the sequence-based network (Fig. S5).

The vast majority of the protein sequences associated with the thioredoxin fold class have only been examined *in silico*, when gene prediction models are applied after the sequencing of a genome; many of the clusters in Fig. 4 have few if any characterized members. For example, one large group of DsbA-like sequences, representing 697 proteins, has only a single member associated with a function: BdbD from *B. subtilis*, a homolog of *E. coli* DsbA [48] that likely performs the same physiological role (Fig. 5D). Another cluster of DsbA-like sequences is without a single member annotated with a function; this cluster is associated with 437 similar yet mysterious sequences, mostly bacterial but also including proteins from fungi, animals, and plants (Fig. 5B). While all of the sequences considered in this analysis can be classified into finer categories using statistical models as shown by the node colors in Fig. 4, this is quite different from associating each protein with a confident *in vitro* or *in vivo* function. Even in well-studied superfamilies like the GSTs, where many proteins have been extensively characterized *in vitro*, there are far more superfamily members that have never been investigated.

### Use of some members of the Trx fold class is restricted to taxonomic subsets

A closer look at the populations of each Trx fold superfamily reveals key differences in the types of organisms that populate each class. By focusing on the species associated with each sequence in the Trx fold class, as summarized in Figures 5–7, it is clear that most superfamilies are dominated by bacterial sequences, both in terms of representative diversity and overall number. Viewing a map of the Trx fold proteins colored by organism type affirms and contextualizes previous knowledge about Trx domain usage in different species (Figures 5–7, Fig. S6). Bacteria and eukaryotes have taken alternate approaches to folding proteins in the periplasm and endoplasmic reticulum, with the bacterial DsbA and DsbC proteins serving as disulfide bond oxidants and isomerases, respectively, while both roles are played by protein disulfide isomerase (PDI) in eukaryotes [49]. The three dimensional structure of yeast PDI has a strikingly similar overall shape compared to the functional DsbC dimer, while still representing a fundamentally different variation of the Trx fold [49]; DsbC has no detectable sequence similarity and a different ordering of secondary structure in comparison with PDIs. The corresponding sequence clusters for DsbA-like superfamily proteins (Fig. 5B–D) and PDI proteins (Fig. 6H) are nearly all bacterial or all eukaryotic. Yet a transition in the phylogenetic class of species expressing a version of the Trx fold is sometimes associated with a change in the biological role for that protein. For example, one sequence cluster associated with the DsbA-like superfamily containing GST kappa (Fig. 5A) has been associated with glutathione transferase activity *in vitro* for two decades [50], but has strong structural similarity to the DsbA-like enzymes [51] (Fig. 5B–D). Unlike the rest of the DsbA-like group, the GST kappa-like enzymes are found in all classes of organisms, and just recently mouse GST kappa was shown to regulate secretion of the adipocyte-derived hormone adiponectin [52]. Likewise, while most types of cytosolic glutathione transferases are found in all types of organisms (Fig. 6J), a number of GST “subgroups” are dominated by eukaryotic organisms (Fig. 6L); many of these GSTs are associated with eukaryote-specific roles such as the biosynthesis of prostaglandins [53] and steroid hormones [54].

Cross-referencing species class and sequence similarity using a network may also be of use in exploring potential drug targets. The network topology indicates that there are many protozoan parasite proteins that are distantly but definitively associated with more familiar classes of human proteins (see Fig. S6). The eukaryote-dominated cytosolic GSTs and PDI-like proteins (Fig. 6L,H) are fringed with loosely connected sequences from protozoan parasites; many of these are distant homologs of human enzymes. (In this work, eukaryotic species not falling into the eukaryotic kingdoms of Metazoa, Fungi, and Viridiplantae are labeled Eukaryota, and due to sampling biases, they are mostly protozoan parasites.) While a number of these proteins are already drug targets (e.g., [12],[55],[56]) this network representation also provides a useful list of additional proteins for consideration; particularly outside of model organisms, few of these proteins have been characterized.

Finally, while some of the sequence groups associated with uniquely eukaryotic biological roles have already been discussed here, the comparative genomics panorama provided by the network implicates other classes of Trx fold proteins in ancient and critical functions such that the fold has been conserved in sequence and structure from prokaryote to animal; these include the classic thioredoxins involved in reduction of ribonucleotide reductase; glutathione peroxidases; the cytosolic GSTs including the omega, zeta, and theta “subgroups”; and the peroxiredoxins (Fig. 6G,K,J, Fig. 7P–Q).

### The Trx fold class exhibits variations on the CxxC active site motif

**Figure 8:**
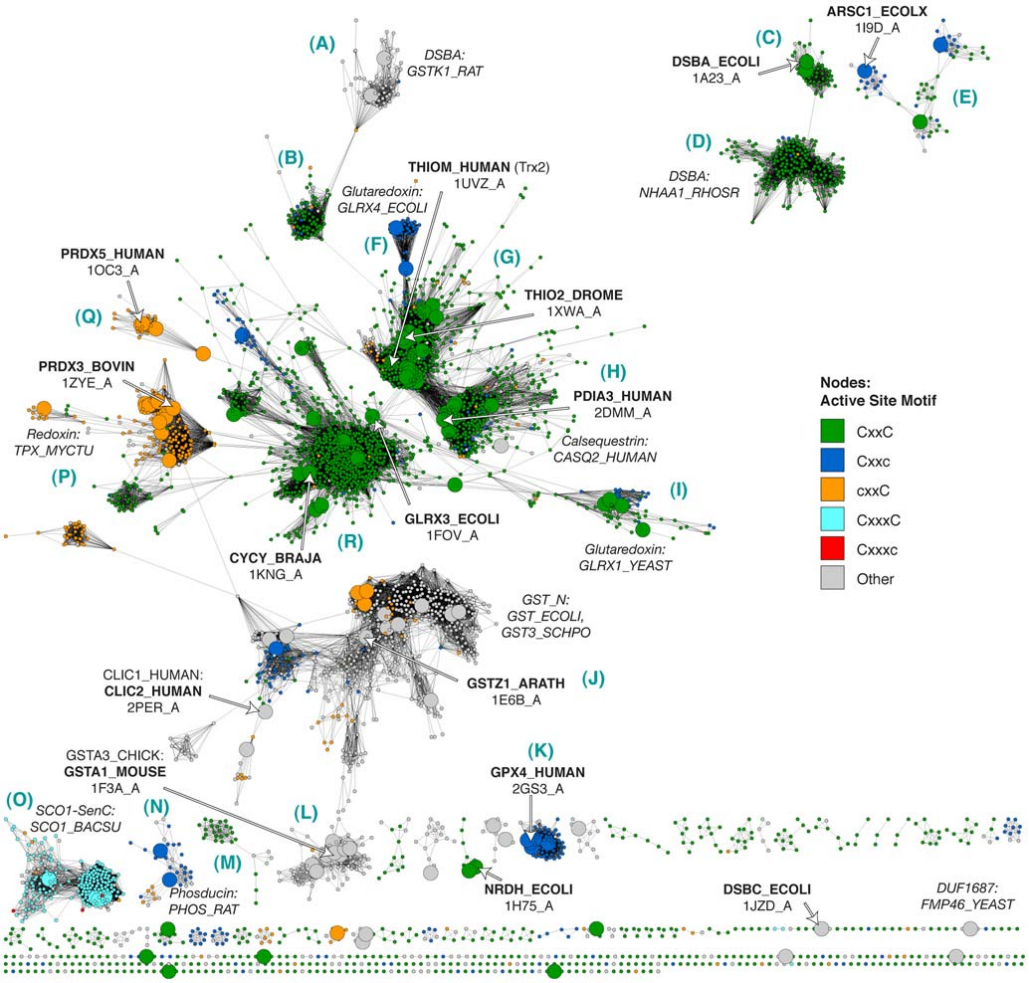
Variations of the CxxC active site are associated with Trx superfamilies. The same sequence similarity network from Fig. 4, containing 4,082 sequences, is colored according to predicted active site architecture. Active site types are abbreviated using a motif like “CxxC”, where a ‘C’ indicates presence of a cysteine, and ‘c’ indicates the presence of some residue other than cysteine. CxxxC means that the two cysteines are present and separated by three amino acids. Examples of each type are shown in Fig. 2. Large nodes represent sequences that are associated with the structures from Fig. 3. Predictions are based on sequence alignments to PFAM Thioredoxin-like Clan HMMs. Cysteines and selenocysteines are treated as equivalent in this figure. Letter labels in blue correspond to sequence groups in Figures 5–7.

To the extent that members of the Trx fold suprafamily have been characterized, some aspect of the residues involved in catalysis invariably occur in the same location relative to the fold. While most sequences in the Trx fold class use two cysteines positioned at the N-terminus of an alpha helix in their catalytic mechanisms (see Fig. 1A), many other catalytic motifs are seen in the fold class, even within superfamilies that are historically associated with the dithiol thioredoxin mechanism. In nearly all Trx fold mechanisms that involve the reduction of a substrate, the first step is a nucleophilic attack by a thiolate from the CxxC motif, typically from the N-terminal cysteine, eventually leading to an oxidized active site that is reduced through a variety of mechanisms to regenerate the active enzyme. Fig. 2 gives examples of the Trx fold active sites categorized by the level of retention of the CxxC motif. In Figures 5–8, these different active site types are mapped onto the sequence network of the Trx fold class, with Figures 5–7 including group-wise depictions of the types of amino acids found at the two key positions of the CxxC motif. These data show that the most common substitution at a CxxC position is cysteine-to-serine or cysteine-to-threonine, depending on the superfamily. Most of the sequences in Fig. 8 contain the archetypal dithiol CxxC motif (56.8% of 4,082). Just 8.9% have just the N-terminal cysteine motif (Cxxc), and 7.6% have just the C-terminal cysteine motif (cxxC). Another 22% of the sequences have none of the Cys-containing motifs from Fig. 2, or are too unusual to estimate an active site.

Most alternative variations of the CxxC active site motif are typified by a specific Trx fold superfamily or subclass. Characterization of the mechanisms in model proteins has been the focus of a great deal of investigation, and the presence of many exceptions to the expected motifs, particularly in classes that contain well-characterized proteins, are surprising and expand on the observations by Fomenko and Gladyshev in model organisms [16],[17]. The Cxxc motif is traditionally associated with the monothiol glutaredoxins (Fig. 5F; mechanism in Fig. 1B); analysis of the variation in that group indicates that the C-terminal position in the motif is typically occupied by a serine, and less frequently by a threonine. Other groups falling into the Cxxc category are the glutathione peroxidases (Fig. 6K); in this case, the C-terminal position is usually a threonine. Some ArsC-type proteins have the Cxxc motif with Ser or Val in the last position, while the SPX-like ArsC proteins have the dithiol CxxC motif. The most common example of the cxxC motif, in which the C-terminal Cys provides the nucleophilic thiolate, is the peroxiredoxins (Fig. 7P,Q). In most Prx-like proteins, this nucleophile is likely stabilized in part by an N-terminal threonine (71.7%)—a role first suggested by Fomenko & Gladyshev [17]—but in 16.3%, an N-terminal serine appears to play this role. Only the SCO1-type proteins exhibit a dithiol motif with two cysteines separated by three residues (Fig. 7O).

In addition to their structural distinctiveness relative to other members of the Trx fold (Fig. 3A), GSTs represent the most populous superfamily that is a poor fit to the CxxC active site motif model. The majority of the 22% of Trx fold sequences in Fig. 8 that do not have a cysteine-containing active site motif (69%) are glutathione transferases. The GST kappa class (Fig. 5A) is actually more like the DsbA-like enzymes in sequence and structure, but the serine found at the N-terminus of the CxxC motif region appears to be critical to its mechanism [51]. Many cytosolic GSTs are associated with a similar catalytic serine [57] (Fig. 6J), but this class is large and heterogeneous and does not fit into the CxxC active site classification as neatly as most of the other Trx fold superfamilies. However, the relatively recently characterized omega GSTs (Fig. 6J: blue nodes) stand out as supporting the Cxxc active site architecture; the N-terminal cysteine has been implicated in the catalytic mechanism of these proteins [11], and their physiological reaction is likely more akin to a glutaredoxin than a canonical glutathione transferase. GST superfamily member yfcG from *E. coli*, which is distantly related to the phi, theta, and beta GST subgroups, efficiently reduces a model glutaredoxin substrate and exhibits an active site threonine at the N-terminal position of the CxxC motif; the side chain is within hydrogen bonding distance of the sulfur of glutathione [43]. The primarily eukaryotic GST class (Fig. 6L), consisting of the alpha, mu, pi, and sigma subgroups, has none of the archetypal Trx fold catalytic machinery at the N-terminus of the first alpha helix in the Trx fold. Thus, from the perspective of structure and catalysis, GSTs are truly a unique constituent of the Trx fold class. One of the next challenges for understanding how function is delivered in the Trx fold class will be to show how the structurally distant GSTs retain and modify aspects of the Trx fold to enable their unique spectrum of catalytic and *in vivo* function.

### A new perspective on the relationship between thioredoxins, cytochrome maturation proteins, and peroxiredoxins

In 2004, Copley and colleagues postulated that peroxiredoxins evolved from a thioredoxin-like ancestor, noting that peroxiredoxins and thioredoxins could be related by sequence and structure using bridging motifs found in the cytochrome maturation proteins (CMP) [58]. These transitive relationships are also seen in the analysis in this work, both from the perspective of sequence and from structure. In terms of sequence similarity, there is a tighter bridge between thioredoxins and CMPs, whereas considering primarily structural information, the relationship between CMPs and peroxiredoxins is closer. Although a large-scale analysis does not provide mechanistic details, incorporating information from the full fold class rather than tracking isolated examples reinforces and contextualizes the significance of the relationship.

**Figure 9:**
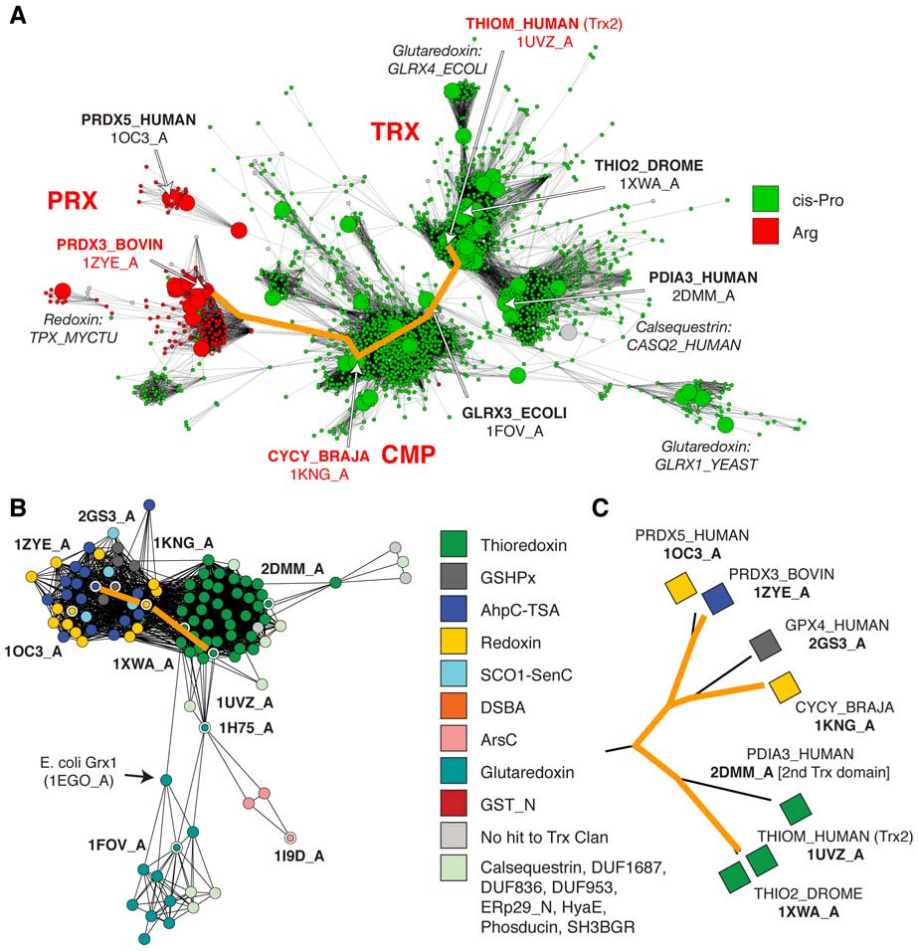
Transitive similarity relationships link the thioredoxins and the peroxiredoxins. **A** Subset of the sequence similarity network from Fig. 4, with nodes colored according to the identity of the amino acid predicted to occcupy the position of the *cis*-proline at the N-terminus of beta strand 3 in the Trx fold (Pro 75 in human Trx 1). The orange path traces transitive sequence similarity relationships between human Trx 2, passing through *B. japonicum* CMP (CYCY_BRAJA), and ending at bovine Prx 3 (PRDX3_BOVIN). Large nodes represent sequences that are associated with the structures from Fig. 3. Predictions are based on sequence alignments to PFAM Thioredoxin-like Clan HMMs. **B** The same path—connecting the structures associated with the sequences in A—traced through a subset of the structure-based network from Fig. 3B. **C** The same path traced through a subset of the structure-based hierarchical clustering of representative structures from Fig. 3D.

There is an unambiguous sequence relationship between the CMPs and thioredoxins. As shown in Fig. 8, these two groups use the CxxC active site. The sequence similarity network in Fig. 9A emphasizes an additional feature: CMPs and thioredoxins contain a *cis*-proline at the N-terminus of the third beta strand (Pro75 in human Trx 1; see Fig. 2A); notably, this proline is more strongly conserved across groups of Trx-fold proteins than the CxxC catalytic dyad. The biophysical function of the *cis*-proline is not well-defined; it likely forms part of the binding site for substrate polypeptides [58] and may serve to prevent metal binding to the CxxC motif [59]. In peroxiredoxins, the *cis*-proline position is occupied by an arginine. Unsurprisingly, the arginine plays a different role: the positively charged side chain is near enough to help lower the pK_a_ of the peroxidatic cysteine, presumably enhancing its nucleophilicity [60].

There is clear structural similarity between peroxiredoxins and thioredoxins, with a representative CMP structure (PDB:1KNG) occupying an intermediate position between the other two classes, while being slightly more similar to the peroxiredoxins (Fig. 9B). This structural similarity is greater than simply sharing variants of the thioredoxin fold: both the CMPs and the peroxiredoxins have an N-terminal extension and an additional insertion between the second beta strand and second alpha helix of the Trx fold (discussed in Results I). Furthermore, the glutathione peroxidases are also structurally intermediate between the peroxiredoxin and thioredoxin groups (Fig. 9C); in fact, the glutathione peroxidases also have a similar N-terminal extension and insertion. Thus peroxiredoxins, glutathione peroxidases, and CMPs are more similar to one another with respect to overall structural similarity and presence of secondary structure elements when compared to thioredoxin. These bridging motifs present in sequence and structure bolster the relationship between thioredoxins and peroxiredoxins, and provide examples of how modifications to the Trx fold correlate with changes in function.

By viewing the peroxiredoxin-thioredoxin relationship from within the context of the entire Trx fold class, we note two new points of interest: First, that it is important to consider glutathione peroxidases as an additional bridging group. From a functional perspective, glutathione peroxidases are a special class of peroxiredoxin; they are structurally more similar to CMPs than other peroxiredoxin classes, and they are also intermediate in structure between the thioredoxins and other peroxiredoxin classes. Second, although all of these groups are quite distant from each other, near or below 30% identity for sequence comparisons between groups, the full landscape of the thioredoxin fold class is much larger and represents more diversity than these three groups.

### Conclusions

The Trx fold class is one of the largest sets of proteins likely to have evolved from a common ancestor, incorporating at least eighteen individual superfamilies and comprising about 0.45% of the entire UniProt sequence database. In this work, we have shown how each protein in the fold class can be viewed from within the context of the features provided by the Trx fold, alongside each other member of the class. What this brings is a new emphasis: here, proteins were compared to the entire population of their class, rather than just to a few well-known archetypal examples. By observing population trends, a new picture has emerged that incorporates more of the real complexity present in the thioredoxin fold class—for example, almost no sequence motif is conserved absolutely—and there is additional information from considering natural groupings of similar proteins rather than reducing protein-protein similarity to closest neighbors. Viewing features of the glutaredoxin-like proteins demonstrates how unusual they are relative to the other major superfamilies: glutaredoxin domains are quite diverse and are found embedded in sequences of dramatically varying lengths and in combination with other domains, indicating an enhanced level of modularity relative to other Trx fold domains. Similarly, glutathione transferases are revealed as especially unique when viewed from the context of the entire Trx fold. While the Trx fold class as a whole is dominated by bacterial sequences, a few groups like protein disulfide isomerases were uniquely present in eukaryotic organisms. Finally, as demonstrated with the cytochrome maturation proteins, the Trx fold context can be used to show how features of one superfamily are either retained or modified in a neighboring superfamily, tracing out a transitive similarity pathway. The Trx fold class is primarily composed of proteins that have no annotated function and have never been investigated *in vitro*. However, identifying where a protein falls within the similarity landscapes described here provides information about basic catalytic capabilities of that protein. Boundaries between functional classes are implicit in the network topologies, and this can inform the characterization of proteins without annotations, as well as expose proteins that may have been misannotated. This analysis provides a working blueprint for predicting the catalytic possibilities of new members of the Trx fold class.

## Methods

### Data set sources and curation

To assemble all sequences from the Trx fold class, the data set consisted of the union of all sequences that were members of the PFAM Thioredoxin-like Clan [28] and all sequences classified into relevant Trx fold superfamilies in SwissProt [9]. Members of the Trx Clan were all sequences from the UniProt Knowledgebase Release 14.0 (7/22/08) [9] that aligned to the PFAM Thioredoxin-like Clan (CL0172) member HMMs (ls model) from PFAM release 22.0 (6/27/07) [27] with a score better than the PFAM gathering threshold. The 20 relevant SwissProt superfamilies are: FMP46 family, GST superfamily, OST3/OST6 family, SCO1/2 family, SH3BGR family, UPF0413 family, ahpC/TSA family, arsC family, calsequestrin family, chloride channel CLIC family, glutaredoxin family, glutathione peroxidase family, hupG/hyaE family, iodothyronine deiodinase family, nucleoredoxin family, peroxiredoxin 2 family, phosducin family, protein disulfide isomerase family, quiescin-sulfhydryl oxidase (QSOX) family, thioredoxin family. This union set of all Trx fold sequences contained 29,206 sequences.

Sequences used in sequence similarity networks were filtered to a maximum of 40% sequence identity using CD-HIT [61]. Additionally, only sequences longer than 60 amino acids were used in the networks, resulting in a data set of 4,082 sequences.

The structures analyzed were the 159 chains associated with the above 29,206 sequences that were not theoretical models and had chain sequences with a maximum of 60% identity to any other chain as determined by CD-HIT.

### Construction of networks: sequence & structure

The sequence similarity networks were constructed as described in Atkinson et al. 2009 [25], with pairwise similarities between proteins determined using pairwise BLAST alignments [62] and resulting networks visualized in Cytoscape 2.6 using the Organic layout [63]. The structure similarity networks were constructed and visualized in the same way, except pairwise similarity between structure chains was determined using FAST [64].

### Construction of hierarchical clustering tree

The pairwise structural similarities from the FAST algorithm were used to construct a tree using hierarchical complete linkage clustering. The tree was visualized in Dendroscope [65].

### Annotations of families and taxonomic categories

This work includes a number of networks and a tree with proteins colored according to a specific type of annotation. Structures were annotated as members of PFAM families if the amino acid sequences from the Protein Data Bank SEQRES records [66] aligned to the PFAM family ls model with a score better than the PFAM gathering threshold. Sequences were annotated as PFAM family members using the same criteria. Sequences were annotated to a SwissProt family (Fig. S2) using the SwissProt SIMILARITY records. Presence of domains in a sequence was assessed using the PFAM family fs models (Fig. S3). Species were assigned to a kingdom or superkingdom using the NCBI taxonomy database [67]. Classification in other databases as listed in Table S4 was determined using SCOP 1.75 (June 2009) [68] and CATH 3.2.0 (July 2008) [69].

### Prediction of CxxC active sites

All CxxC active site motifs were located using representative structures, and the corresponding motif was identified in each PFAM Trx Clan ls HMM. The amino acids aligning to this motif in the HMM were used to determine the active site motif for each sequence. See supplementary data website for specific motifs based on structural information.

### External supplementary data website

All data files generated in the analysis, including sequence files and networks, are available online at http://babbittlab.compbio.ucsf.edu/resources/TrxFold. Figures including similarity networks are static representations of interactive network files that can be downloaded from the website and manipulated using Cytoscape.

## Supporting Information

Figure S1 A structure-based similarity network describes a map of the Trx fold class: colored by minority Thioredoxin-like Clan families. A Structure similarity network, containing 159 structures that are a maximum of 60% identical (by sequence) that span the Trx fold class. Similarity is defined by FAST scores better than a score of 4.5; edges at this limiting score represent alignments with a median of 2.75 Å RMSD across 72 aligned positions. Each node is colored by a PFAM Thioredoxin-like Clan family if the chain sequence is a member of that family. Nodes with thick red borders and bold labels denote chains present in the hierarchical clustering tree in D. Labels like “1ON4_A” denote PDB ID 1ON4, chain A. B Structure similarity network containing the same structures as in A, shown at the more stringent threshold of 7.5. Edges at this limiting score correspond to alignments with a median of 2.45 Å RMSD across 89 aligned positions. Nodes are colored as in A. C Structure similarity network containing the 105 structures from the large connected cluster in B, displayed at a FAST score cutoff of 12.0; edges at this limiting score represent alignments with a median of 2.21 Å RMSD across 102 aligned positions. Nodes are colored as in A. D Complete linkage hierarchical clustering tree based on pairwise FAST scores for 15 representative structures singled out in the networks in A–C, with PDB IDs in bold, and associated SwissProt sequence IDs in plain text.
(1.92 MB TIF)

Figure S2 Sequence similarity network, containing 4,082 representative sequences that are a maximum of 40% identical that span the Trx fold class. Similarity is defined by pairwise BLAST alignments better than an E-value of 1×10−12; edges at this threshold represent alignments with a median 30% identity over 120 residues, while the rest of the edges represent better alignments. Each node is colored by the sequence's SwissProt family classification, if available; sequences that are not classified in SwissProt are colored grey. Large nodes represent sequences that are at least 40% identical to the 159 structures in Fig. 3. The sequences associated with the 15 representative structures in Fig. 3C are labeled using bold text and white arrows. The general locations of other sequences representing different superfamilies are noted using italicized text.
(1.79 MB TIF)

Figure S3 Many Trx domains occur in combination with other Trx domains. A Sequence similarity network, containing 4,082 representative sequences that are a maximum of 40% identical that span the Trx fold class. Similarity is defined by pairwise BLAST alignments better than an E-value of 1×10−12; edges at this threshold represent alignments with a median 30% identity over 120 residues, while the rest of the edges represent better alignments. Nodes are colored by the number of PFAM Thioredoxin-like Clan family domains occurring within the sequence; with the exception of H. influenzae Prx 5–labeled (iii)–and the monothiol glutaredoxins–labeled (ii)–these domains are typically duplications of the same domain, such as the PDI-type enzymes (iv), which can contain two to four thioredoxin domains, or the few DSBA-like enzymes (i) which contain up to three DSBA-like domains. Large nodes represent sequences that are at least 40% identical to the 159 structures in Fig. 3. The sequences associated with the 15 representative structures in Fig. 3C are labeled using bold text and white arrows. The occurrence of other sequences representing different superfamilies are noted using italicized text. B Domain structures for example sequences from the groups labeled (i)–(iv); some domains are shorter than expected and this is denoted by a gradient that fades to white. The sequences are identified by their UniProt sequence IDs.
(1.77 MB TIF)

Figure S4 The relative populations of the Trx fold superfamilies vary. A 4,082 representative sequences that are a maximum of 40% identical and span the Trx fold class, binned according to their membership in PFAM families within the Thioredoxin-like Clan. B All 29,206 sequences in the Trx fold class.
(0.54 MB TIF)

Figure S5 There is good correspondence between the structure and sequence-based Trx fold class networks. The three views of the structure-based network from Fig. 3 are repeated in A–C, and panel D contains a sequence-based network derived from the amino acid sequences in the 159 structure chains. A Structure similarity network, containing 159 structures that are a maximum of 60% identical (by sequence) that span the Trx fold class. Similarity is defined by FAST scores better than a score of 4.5; edges at this threshold represent alignments with a median of 2.75A RMSD across 72 aligned positions, while the rest of the edges represent better alignments. Each node is colored by a PFAM Thioredoxin-like Clan family if the chain sequence is a member. Nodes with thick white borders and bold labels denote chains present in the hierarchical clustering tree in Fig. 3D. Labels like “1ON4_A” denote PDB ID 1ON4, chain A. B Structure similarity network containing the same structures as in A, shown at the more stringent threshold of 7.5. Edges at this threshold correspond to alignments with a median of 2.45A RMSD across 89 aligned positions. Nodes are colored as in A. C Structure similarity network containing the 105 structures from the large connected cluster in B, displayed at a FAST score cutoff of 12.0; edges at this threshold represent alignments with a median of 2.21A RMSD across 102 aligned positions. Nodes are colored as in A. D Sequence similarity network, containing 159 chain sequences from A–C. Similarity is defined by pairwise BLAST alignments better than an E-value of 1×10^−5^; edges at this threshold represent alignments with a median 27% identity over 84 residues, while the rest of the edges represent better alignments.
(2.31 MB TIF)

Figure S6 Use of some members of the Trx fold class is restricted to taxonomic subsets. Here, the sequence similarity network from Fig. 4, containing 4,082 sequences, is colored by the species kingdom (Metazoa, Fungi, Viridiplantae) or superkingdom (Bacteria, Eukaryota, Archaea). Note that “Eukaryota” includes all eukyaryotic species without a more specific kingdom, and is primarily associated with protozoan parasites. Large nodes represent sequences that are associated with the structures from Fig. 3. Blue letter labels correspond to sequence groups in Figures 5–7.
(1.96 MB TIF)

Table S1 Number of unique structures in each Thioredoxin-like Clan family
(0.04 MB DOC)

Table S2 Number of sequences in each Thioredoxin-like Clan family
(0.04 MB DOC)

Table S3 Network edges from Fig. 4 due to sequence similarity outside of the domain of interest
(0.03 MB DOC)

Table S4 Mapping between Fig. 5 groups and the databases PFAM, SCOP, and CATH
(0.05 MB DOC)
