# Supplementary figures and images for: An Atlas of the Thioredoxin Fold Class Reveals the Complexity of Function-Enabling Adaptations
Source: PLoS Comput Biol. 2009 Oct 23;5(10):e1000541. doi: 10.1371/journal.pcbi.1000541 (PMC2757866; doi:10.1371/journal.pcbi.1000541)

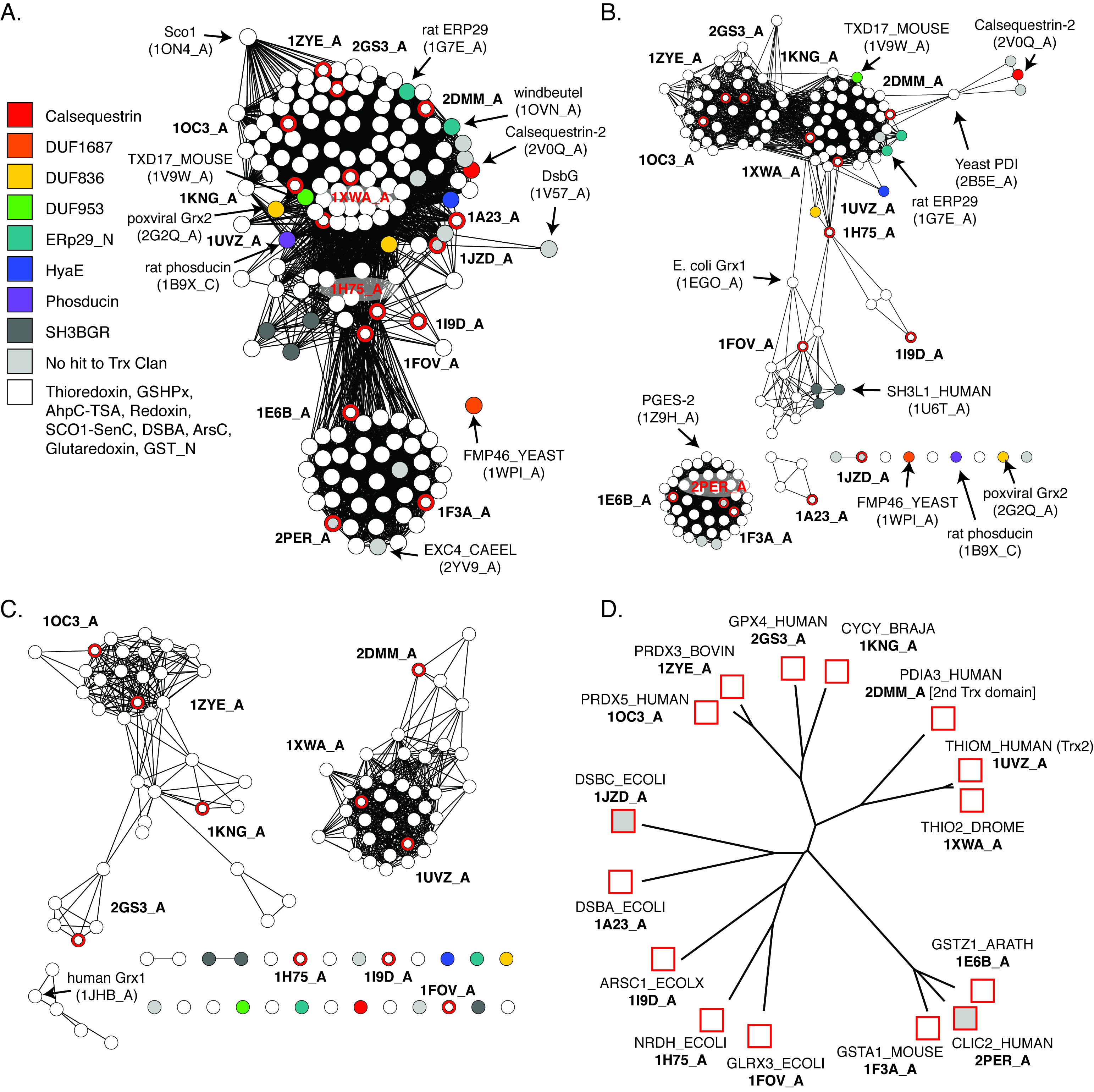

Supplement: Figure S1 — A structure-based similarity network describes a map of the Trx fold class: colored by minority Thioredoxin-like Clan families. A Structure similarity network, containing 159 structures that are a maximum of 60% identical (by sequence) that span the Trx fold class. Similarity is defined by FAST scores better than a score of 4.5; edges at this limiting score represent alignments with a median of 2.75 Å RMSD across 72 aligned positions. Each node is colored by a PFAM Thioredoxin-like Clan family if the chain sequence is a member of that family. Nodes with thick red borders and bold labels denote chains present in the hierarchical clustering tree in D. Labels like “1ON4_A” denote PDB ID 1ON4, chain A. B Structure similarity network containing the same structures as in A, shown at the more stringent threshold of 7.5. Edges at this limiting score correspond to alignments with a median of 2.45 Å RMSD across 89 aligned positions. Nodes are colored as in A. C Structure similarity network containing the 105 structures from the large connected cluster in B, displayed at a FAST score cutoff of 12.0; edges at this limiting score represent alignments with a median of 2.21 Å RMSD across 102 aligned positions. Nodes are colored as in A. D Complete linkage hierarchical clustering tree based on pairwise FAST scores for 15 representative structures singled out in the networks in A–C, with PDB IDs in bold, and associated SwissProt sequence IDs in plain text. (1.92 MB TIF) [file pcbi.1000541.s001.tif]

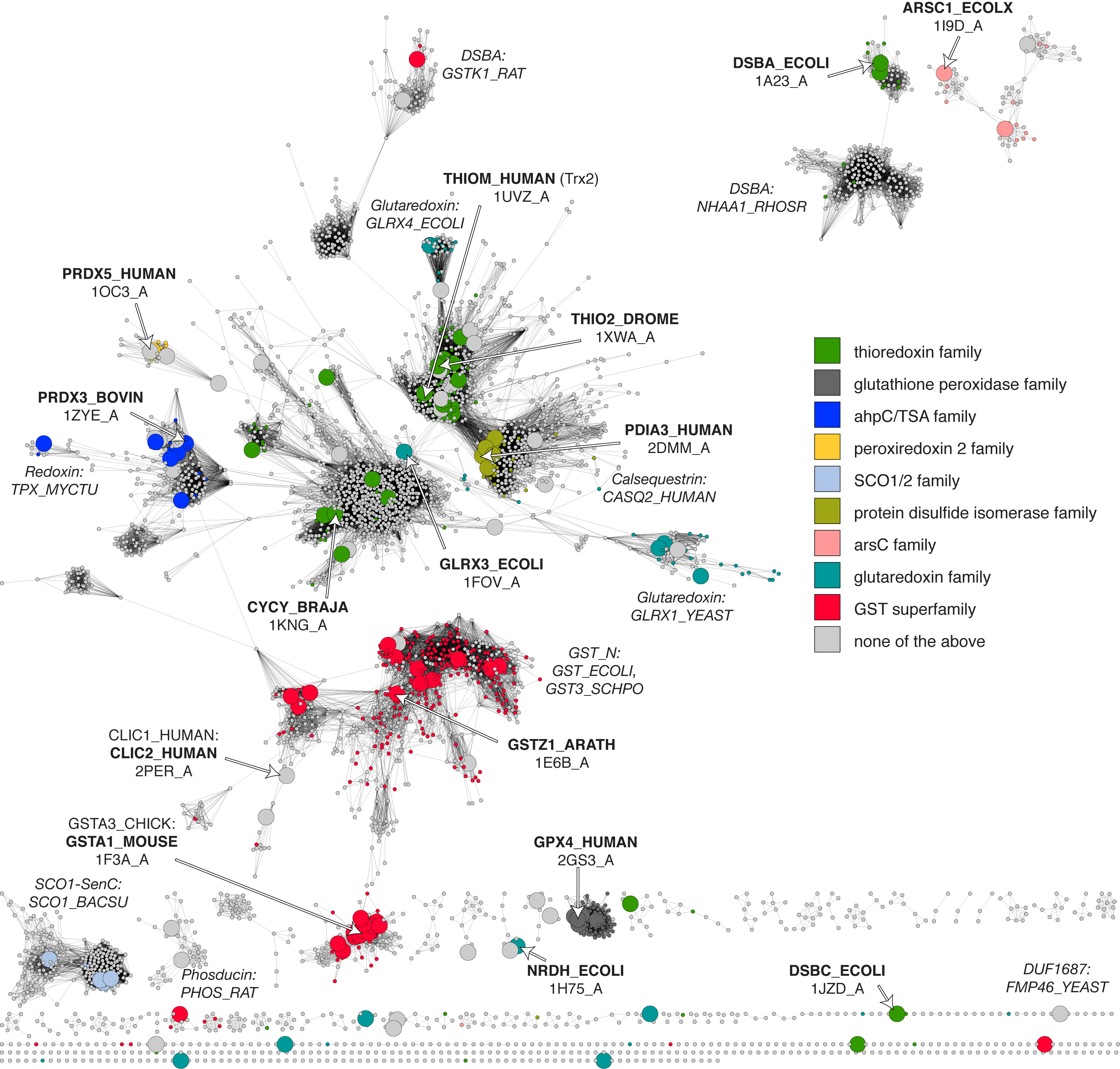

Supplement: Figure S2 — Sequence similarity network, containing 4,082 representative sequences that are a maximum of 40% identical that span the Trx fold class. Similarity is defined by pairwise BLAST alignments better than an E-value of 1×10^−12^; edges at this threshold represent alignments with a median 30% identity over 120 residues, while the rest of the edges represent better alignments. Each node is colored by the sequence's SwissProt family classification, if available; sequences that are not classified in SwissProt are colored grey. Large nodes represent sequences that are at least 40% identical to the 159 structures in Fig. 3. The sequences associated with the 15 representative structures in Fig. 3C are labeled using bold text and white arrows. The general locations of other sequences representing different superfamilies are noted using italicized text. (1.79 MB TIF) [file pcbi.1000541.s002.tif]

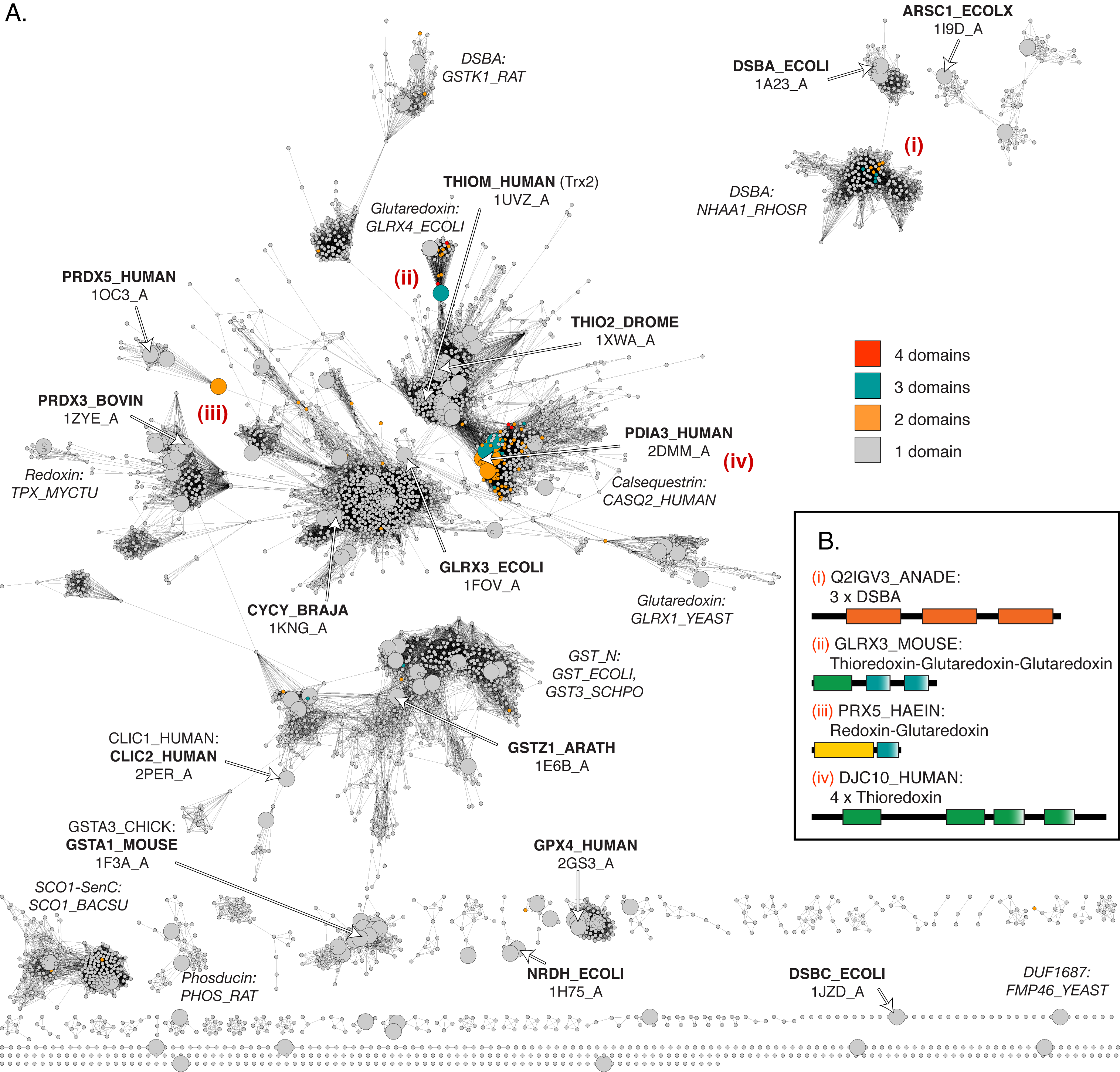

Supplement: Figure S3 — Many Trx domains occur in combination with other Trx domains. A Sequence similarity network, containing 4,082 representative sequences that are a maximum of 40% identical that span the Trx fold class. Similarity is defined by pairwise BLAST alignments better than an E-value of 1×10^−12^; edges at this threshold represent alignments with a median 30% identity over 120 residues, while the rest of the edges represent better alignments. Nodes are colored by the number of PFAM Thioredoxin-like Clan family domains occurring within the sequence; with the exception of H. influenzae Prx 5–labeled (iii)–and the monothiol glutaredoxins–labeled (ii)–these domains are typically duplications of the same domain, such as the PDI-type enzymes (iv), which can contain two to four thioredoxin domains, or the few DSBA-like enzymes (i) which contain up to three DSBA-like domains. Large nodes represent sequences that are at least 40% identical to the 159 structures in Fig. 3. The sequences associated with the 15 representative structures in Fig. 3C are labeled using bold text and white arrows. The occurrence of other sequences representing different superfamilies are noted using italicized text. B Domain structures for example sequences from the groups labeled (i)–(iv); some domains are shorter than expected and this is denoted by a gradient that fades to white. The sequences are identified by their UniProt sequence IDs. (1.77 MB TIF) [file pcbi.1000541.s003.tif]

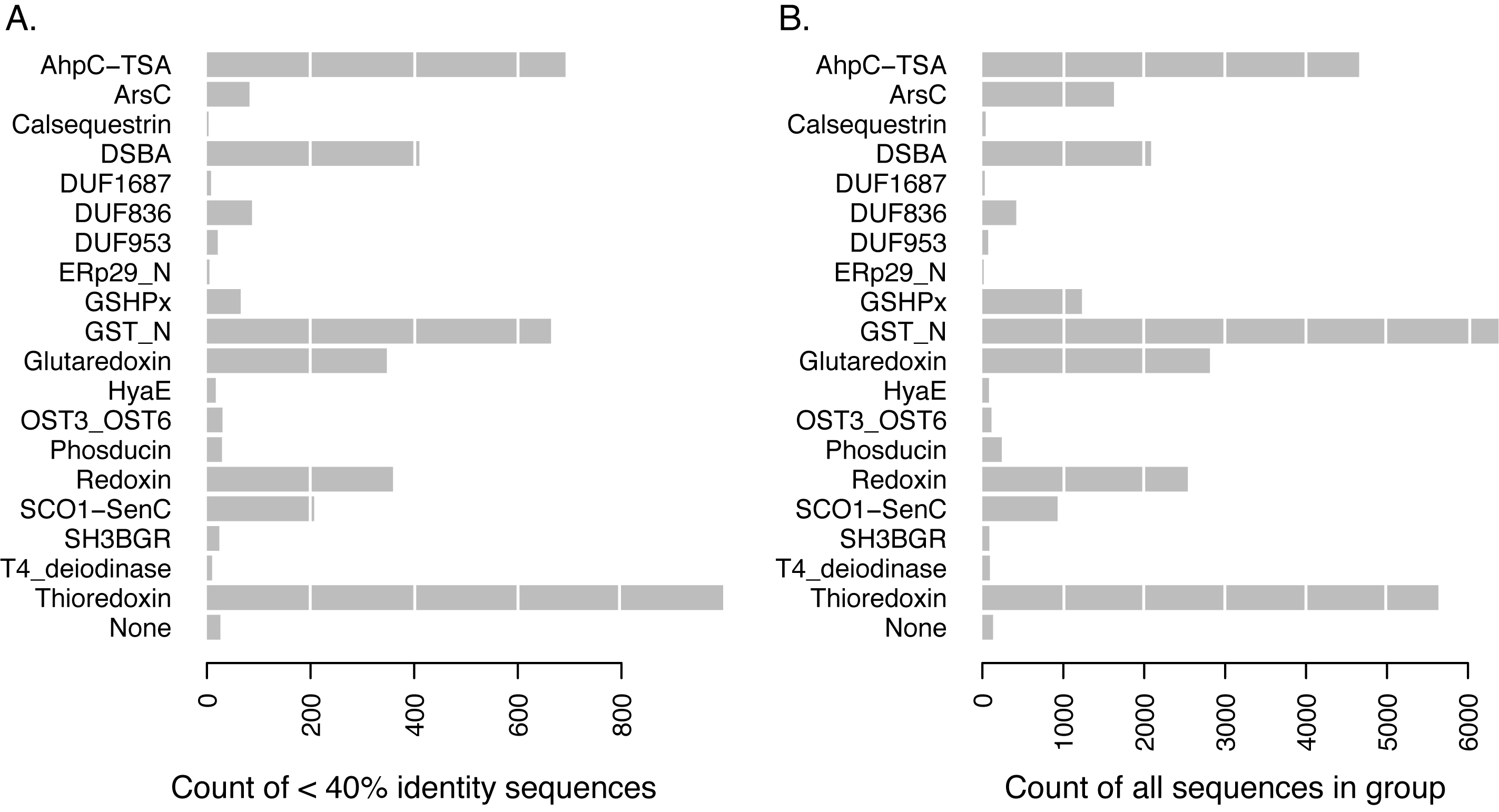

Supplement: Figure S4 — The relative populations of the Trx fold superfamilies vary. A 4,082 representative sequences that are a maximum of 40% identical and span the Trx fold class, binned according to their membership in PFAM families within the Thioredoxin-like Clan. B All 29,206 sequences in the Trx fold class. (0.54 MB TIF) [file pcbi.1000541.s004.tif]

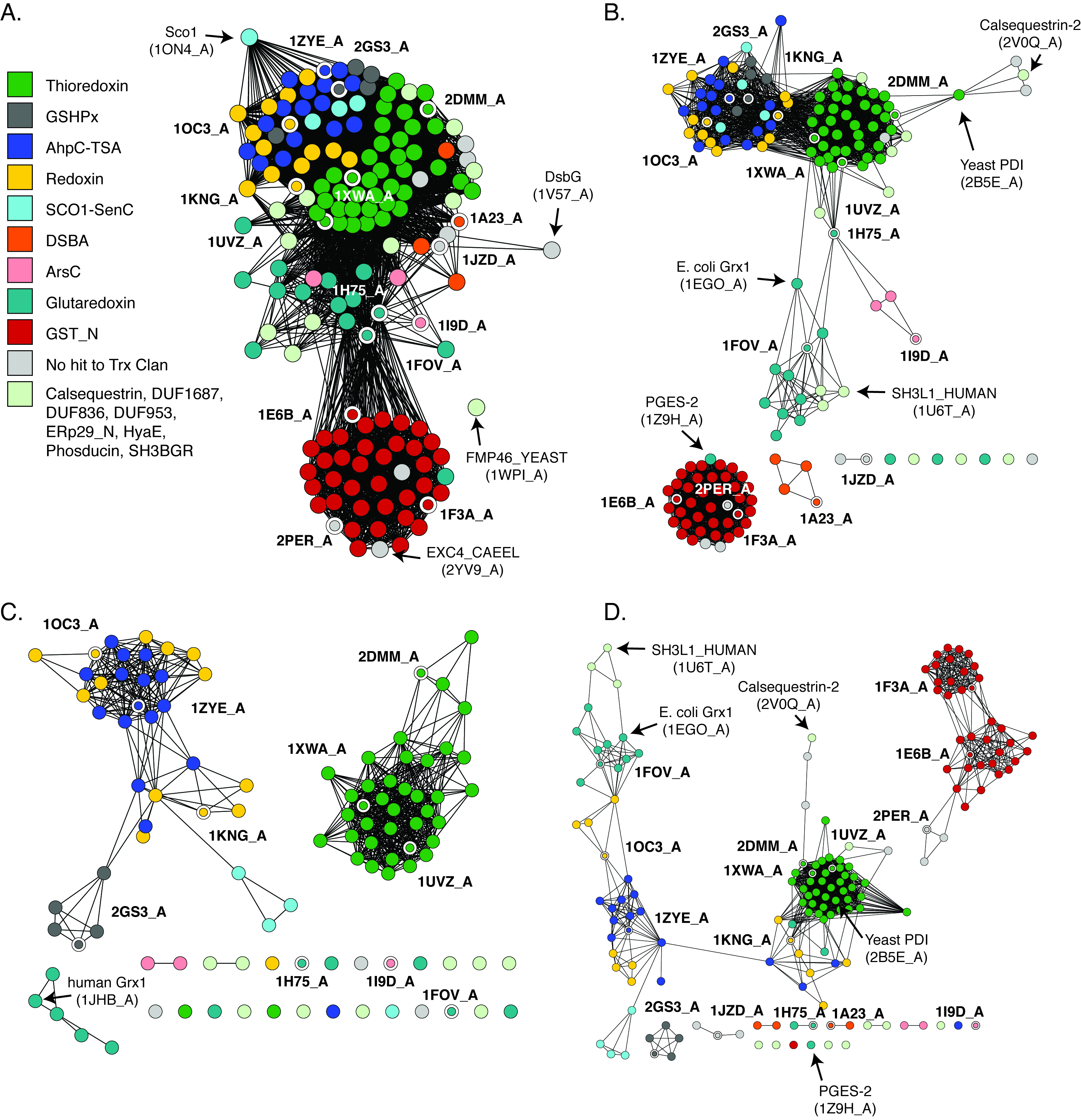

Supplement: Figure S5 — There is good correspondence between the structure and sequence-based Trx fold class networks. The three views of the structure-based network from Fig. 3 are repeated in A–C, and panel D contains a sequence-based network derived from the amino acid sequences in the 159 structure chains. A Structure similarity network, containing 159 structures that are a maximum of 60% identical (by sequence) that span the Trx fold class. Similarity is defined by FAST scores better than a score of 4.5; edges at this threshold represent alignments with a median of 2.75A RMSD across 72 aligned positions, while the rest of the edges represent better alignments. Each node is colored by a PFAM Thioredoxin-like Clan family if the chain sequence is a member. Nodes with thick white borders and bold labels denote chains present in the hierarchical clustering tree in Fig. 3D. Labels like “1ON4_A” denote PDB ID 1ON4, chain A. B Structure similarity network containing the same structures as in A, shown at the more stringent threshold of 7.5. Edges at this threshold correspond to alignments with a median of 2.45A RMSD across 89 aligned positions. Nodes are colored as in A. C Structure similarity network containing the 105 structures from the large connected cluster in B, displayed at a FAST score cutoff of 12.0; edges at this threshold represent alignments with a median of 2.21A RMSD across 102 aligned positions. Nodes are colored as in A. D Sequence similarity network, containing 159 chain sequences from A–C. Similarity is defined by pairwise BLAST alignments better than an E-value of 1×10^−5^; edges at this threshold represent alignments with a median 27% identity over 84 residues, while the rest of the edges represent better alignments. (2.31 MB TIF) [file pcbi.1000541.s005.tif]

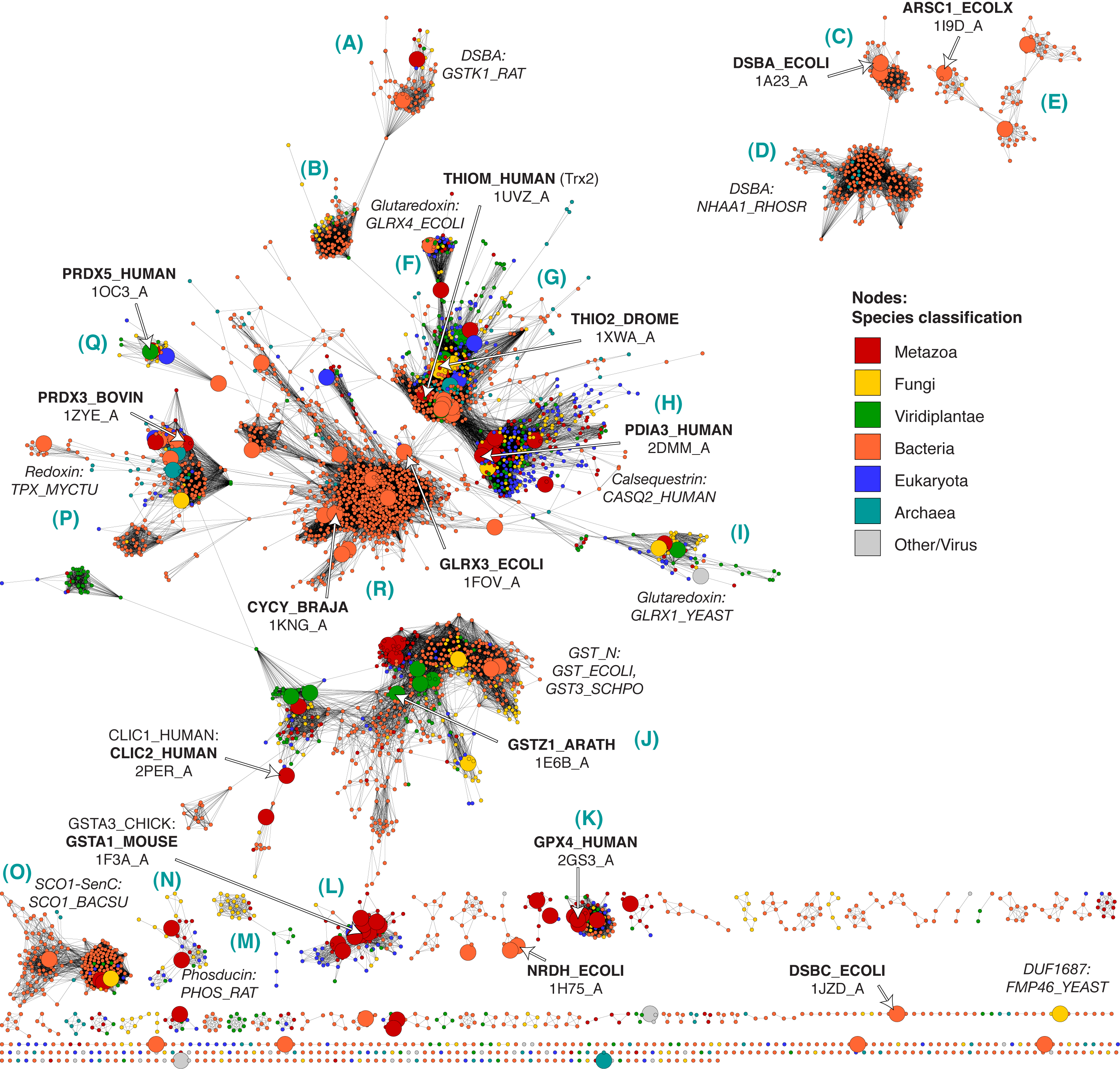

Supplement: Figure S6 — Use of some members of the Trx fold class is restricted to taxonomic subsets. Here, the sequence similarity network from Fig. 4, containing 4,082 sequences, is colored by the species kingdom (Metazoa, Fungi, Viridiplantae) or superkingdom (Bacteria, Eukaryota, Archaea). Note that “Eukaryota” includes all eukyaryotic species without a more specific kingdom, and is primarily associated with protozoan parasites. Large nodes represent sequences that are associated with the structures from Fig. 3. Blue letter labels correspond to sequence groups in Figures 5– 7. (1.96 MB TIF) [file pcbi.1000541.s006.tif]
