# Supplementary material for: An Atlas of the Thioredoxin Fold Class Reveals the Complexity of Function-Enabling Adaptations
Source: PLoS Comput Biol. 2009 Oct 23;5(10):e1000541. doi: 10.1371/journal.pcbi.1000541 (PMC2757866; doi:10.1371/journal.pcbi.1000541)
Supplement: Table S1 — Number of unique structures in each Thioredoxin-like Clan family (0.04 MB DOC) [file pcbi.1000541.s007.doc]

### Table S1. Number of unique structures in each Thioredoxin-like Clan family

| **PFAM model** | **Sequences with structures^1^** | **Count of structures** |
| --- | --- | --- |
| AhpC-TSA | 24 | 42 |
| ArsC | 4 | 13 |
| Calsequestrin | 3 | 4 |
| DSBA | 5 | 24 |
| DUF1687 | 1 | 1 |
| DUF836 | 2 | 3 |
| DUF953 | 2 | 2 |
| ERp29_N | 2 | 6 |
| GSHPx | 7 | 8 |
| GST_N | 67 | 216 |
| Glutaredoxin | 18 | 30 |
| HyaE | 3 | 3 |
| OST3_OST6 | 0 | 0 |
| Phosducin | 2 | 4 |
| Redoxin | 16 | 22 |
| SCO1-SenC | 4 | 15 |
| SH3BGR | 4 | 6 |
| T4_deiodinase | 0 | 0 |
| Thioredoxin | 41 | 131 |
| None^2^ | 12 | 33 |
| Total | 217 | 563 |

^1^For each PDB ID, a structure is counted once for each unique chain sequence containing a Trx fold.

^2^33 chains included in this analysis exhibited a Trx fold, but did not align to a PFAM Thioredoxin-like Clan model with a score better than the gathering threshold.
