# Supplementary material for: An Atlas of the Thioredoxin Fold Class Reveals the Complexity of Function-Enabling Adaptations
Source: PLoS Comput Biol. 2009 Oct 23;5(10):e1000541. doi: 10.1371/journal.pcbi.1000541 (PMC2757866; doi:10.1371/journal.pcbi.1000541)
Supplement: Table S2 — Number of sequences in each Thioredoxin-like Clan family (0.04 MB DOC) [file pcbi.1000541.s008.doc]

### Table S2. Number of sequences in each Thioredoxin-like Clan family

| PFAM **model** | **Max 40% identical sequences^1^** | **All sequences** |
| --- | --- | --- |
| AhpC-TSA | 692 | 4,655 |
| ArsC | 82 | 1,626 |
| Calsequestrin | 3 | 40 |
| DSBA | 410 | 2,085 |
| DUF1687 | 8 | 29 |
| DUF836 | 87 | 419 |
| DUF953 | 21 | 71 |
| ERp29_N | 5 | 16 |
| GSHPx | 65 | 1,230 |
| GST_N | 664 | 6,378 |
| Glutaredoxin | 347 | 2,811 |
| HyaE | 17 | 81 |
| OST3_OST6 | 30 | 112 |
| Phosducin | 29 | 239 |
| Redoxin | 359 | 2,539 |
| SCO1-SenC | 207 | 929 |
| SH3BGR | 24 | 86 |
| T4_deiodinase | 10 | 93 |
| Thioredoxin | 996 | 5,635 |
| None^2^ | 26 | 132 |
| Total | 4,082 | 29,206 |

^1^The total of 29,206 sequences with a Trx fold (see Methods) can be reduced to 4,082 sequences that are longer than 60 amino acids and are no more than 40% identical.

^2^132 Trx fold sequences included in this analysis did not align to a PFAM Thioredoxin-like Clan model with a score better than the gathering threshold.
