# Supplementary material for: An Atlas of the Thioredoxin Fold Class Reveals the Complexity of Function-Enabling Adaptations
Source: PLoS Comput Biol. 2009 Oct 23;5(10):e1000541. doi: 10.1371/journal.pcbi.1000541 (PMC2757866; doi:10.1371/journal.pcbi.1000541)
Supplement: Table S3 — Network edges from Fig. 4 due to sequence similarity outside of the domain of interest (0.03 MB DOC) [file pcbi.1000541.s009.doc]

### Table S3. Network edges from Fig. 4 due to sequence similarity outside of the domain of interest

| **Additional domain responsible for similarity** | **Anchoring group 1** | **Anchoring group 2** |
| --- | --- | --- |
| Kinase | Single GST (Fig. 6J) | Single peroxiredoxin (Fig. 7P); uncharacterized Grx |
| Peptide methionine sulfoxide reductase | Cytochrome maturation-like proteins (Fig. 7R) | Glutaredoxins (Fig. 6I) |
| NB-ARC (ATP-binding domain shared by plant resistance gene produces, cell death regulators in animals) | Single DsbA-like protein (Fig. 5B) | Single Thioredoxin-like protein (Fig. 6G) |
| Glutaredoxin^1^ | Atypical Prx PRX5_HAEIN (Fig. 7Q) | Glutaredoxin GLRX3_ECOLI (~Fig. 6I) |

^1^In this case, the atypical peroxiredoxin from *H. influenzae* is fused to a glutaredoxin domain (PRX5_HAEIN). The peroxiredoxin domain is connected by many edges to the other atypical peroxiredoxins, and there is an additional edge representing a significant alignment between just the glutaredoxin domain of PRX5_HAEIN and *E. coli* Grx 3.
