# Supplementary material for: An Atlas of the Thioredoxin Fold Class Reveals the Complexity of Function-Enabling Adaptations
Source: PLoS Comput Biol. 2009 Oct 23;5(10):e1000541. doi: 10.1371/journal.pcbi.1000541 (PMC2757866; doi:10.1371/journal.pcbi.1000541)
Supplement: Table S4 — Mapping between Fig. 5 groups and the databases PFAM, SCOP, and CATH (0.05 MB DOC) [file pcbi.1000541.s010.doc]

### Table S4. Mapping between Fig. 5 groups and the databases PFAM, SCOP, and CATH

| **Group** | **PFAM family** | **SCOP** | **CATH^1^** |
| --- | --- | --- | --- |
| A. GST kappa | DSBA | Fold: Thioredoxin fold  Superfamily: Thioredoxin-like  Family: DsbA-like | None |
| B. DsbA | DSBA | None | None |
| C. DsbA | DSBA | Fold: Thioredoxin fold  Superfamily: Thioredoxin-like  Family: DsbA-like | T: 3.40.30 Glutaredoxin  H: 3.40.30.10 Glutaredoxin |
| D. BdbD | DSBA | None | None |
| E. ArsC | ArsC | Fold: Thioredoxin fold  Superfamily: Thioredoxin-like  Family: ArsC-like | None |
| F. monothiol Grx | Glutaredoxin, Thioredoxin | Fold: Thioredoxin fold  Superfamily: Thioredoxin-like  Family: Thioltransferase | T: 3.40.30 Glutaredoxin  H: 3.40.30.10 Glutaredoxin |
| G. Thioredoxin | Thioredoxin | Fold: Thioredoxin fold  Superfamily: Thioredoxin-like  Family: Thioltransferase | T: 3.40.30 Glutaredoxin  H: 3.40.30.10 Glutaredoxin |
| H. PDI | Thioredoxin, Calsequestrin, ERp29 | Fold: Thioredoxin fold  Superfamily: Thioredoxin-like  Family: PDI-like | T: 3.40.30 Glutaredoxin  H: 3.40.30.10 Glutaredoxin |
| I. Glutaredoxin | Glutaredoxin | Fold: Thioredoxin fold  Superfamily: Thioredoxin-like  Family: Thioltransferase | T: 3.40.30 Glutaredoxin  H: 3.40.30.10 Glutaredoxin |
| J. GST | GST_N | Fold: Thioredoxin fold  Superfamily: Thioredoxin-like  Family: Glutathione S-transferase (GST), N-terminal domain | **Trx-like domain:**  T: 3.40.30 Glutaredoxin  H: 3.40.30.10 Glutaredoxin  **C-term domain:**  T: 1.20.1050 Glutathione S-transferase Yfyf (Class Pi); Chain A, domain 2  H: 1.20.1050.10 |
| K. GSHPx | GSHPx | Fold: Thioredoxin fold  Superfamily: Thioredoxin-like  Family: Glutathione peroxidases-like | T: 3.40.30 Glutaredoxin  H: 3.40.30.10 Glutaredoxin |
| L. GST | GST_N | Fold: Thioredoxin fold  Superfamily: Thioredoxin-like  Family: Glutathione S-transferase (GST), N-terminal domain | **Trx-like domain:**  T: 3.40.30 Glutaredoxin  H: 3.40.30.10 Glutaredoxin  **C-term domain:**  T: 1.20.1050 Glutathione S-transferase Yfyf (Class Pi); Chain A, domain 2  H: 1.20.1050.10 |
| M. OST3/6 | OST3_OST6 | None | None |
| N. Phosducin | Phosducin, Thioredoxin | Fold: Thioredoxin fold  Superfamily: Thioredoxin-like  Family: Phosducin | T: 3.40.30 Glutaredoxin  H: 3.40.30.10 Glutaredoxin |
| O. Sco1-SenC | SCO1-SenC | Fold: Thioredoxin fold  Superfamily: Thioredoxin-like  Family: Glutathione peroxidases-like | T: 3.40.30 Glutaredoxin  H: 3.40.30.10 Glutaredoxin |
| P. Typical Prx | AhPC-TSA,  Redoxin | Fold: Thioredoxin fold  Superfamily: Thioredoxin-like  Family: Glutathione peroxidases-like | T: 3.40.30 Glutaredoxin  H: 3.40.30.10 Glutaredoxin |
| Q. Atypical Prx | Redoxin | Fold: Thioredoxin fold  Superfamily: Thioredoxin-like  Family: Glutathione peroxidases-like | T: 3.40.30 Glutaredoxin  H: 3.40.30.10 Glutaredoxin |
| R. CMP/DsbE | Redoxin,  AhpC-TSA,  Thioredoxin | Fold: Thioredoxin fold  Superfamily: Thioredoxin-like  Family: Glutathione peroxidases-like | T: 3.40.30 Glutaredoxin  H: 3.40.30.10 Glutaredoxin |

^1^CATH: included annotations are T: Topology, and H: Homologous superfamily.
